# Coal fly ash industrial waste-derived products: a review on the extraction of aluminum and silicon for nanoparticle synthesis

**DOI:** 10.1007/s11356-025-37298-z

**Published:** 2026-01-16

**Authors:** Ntombiphumile Perceverence Tenza, Mathibela Elias Aphane

**Affiliations:** University of South Africa - Florida Campus, Roodepoort, Gauteng South Africa

**Keywords:** Coal fly ash, Waste valorization, Aluminum, Silicon, Extraction, Nanoparticles

## Abstract

C oal fly ash (CFA), a common industrial by-product, poses both environmental challenges and opportunities as a secondary source of silicon and aluminum. This review critically evaluates extraction methods for these elements, focusing on efficiency, selectivity, and scalability. Sintering techniques enhance aluminum recovery by decomposing stable aluminosilicate phases but generally fail to extract silicon, requiring high energy input and large chemical volumes and generating substantial secondary waste. Direct acid leaching selectively dissolves aluminum, often achieving extraction efficiencies above 80%, though crystalline phases such as mullite remain largely unreactive. Alkaline leaching and alkali fusion improve solubilization of both aluminum and silicon, particularly targeting refractory quartz and mullite, but frequently favor silicate recovery and involve significant reagent consumption. Hybrid and combined approaches, including sequential acid–alkali leaching and hydrothermal treatments, achieve more balanced recovery of Al and Si, with moderate to high efficiencies (up to 98%), and reduce energy and chemical demand. Despite progress, most recoveries are still moderate, impurities persist, and scalable, standardized protocols are lacking. While CFA-derived silica nanoparticles are increasingly studied, sustainable alumina nanoparticle synthesis and broader applications remained underexplored. Limited research in regions such as South Africa further restricts local adaptation. Thus, the review highlights the importance of integrated, eco-friendly, and region-specific strategies, reporting both elemental and oxide-based data to enable meaningful comparison and guide future CFA valorization.

## Introduction

Coal fly ash (CFA), a by-product of coal combustion in thermal power plants, has become a significant global environmental challenge. Major coal-dependent nations, including China, India, the United States of America (USA), and Australia, generate hundreds of millions of tons of CFA annually (Chen et al. 2024). China is the largest producer of CFA, generating over 550 million tons annually, yet only around 30% is recycled, primarily through long-established applications in the construction industry as well as environmental uses (Marinina et al. 2021; Lu et al. 2022; Zhao et al. 2023). India follows with approximately 227 million tons annually, much of which is similarly utilized in traditional construction (cement, bricks, and ceramics), road infrastructure, and agricultural applications (Surabhi 2017; Singh et al. 2021; Lu et al. 2022). The USA and Australia have also made progress in CFA valorization, historically employing it in cement production, mine backfilling, and road construction, with more recent interest in its use as a source of alumina and silica (Ahmaruzzaman 2010; Wang et al. 2024). In South Africa, coal remains central to energy production, contributing 83% of total electricity generation in 2023/24 (Fitzgerald et al. 2024). Eskom, South Africa’s national power utility, produces about 34 million tons of CFA annually (Eskom 2024). However, only 7–10% of this ash is recycled, primarily in construction (Vilakazi et al. 2022; Aphane et al. 2024). The remainder is disposed of in ash dumps, raising serious environmental and land-use concerns.

While countries like China and India have diversified the applications of CFA, including its use in cement and concrete production, soil stabilization for roads, embankments, ash dyke raising, landfill liners and covers, mine backfilling, bricks, tiles, inorganic fibers, zeolites, geopolymers, ceramics, fertilizers, and environmental remediation (Marinina et al. 2021; Koshlak 2023; Zhao et al. 2023), South Africa remains largely confined to traditional uses. These include cement, concrete, bricks, blocks, road construction, and mine backfilling, with far less diversification compared to global practices (Vilakazi et al. 2022). This clear contrast illustrates the uneven technological maturity and policy support for CFA valorization worldwide. The limited diversification of CFA utilization in South Africa points to policy, technological, and market gaps that must be addressed to enable value-added applications similar to those adopted internationally.

Because of its underutilization, CFA poses substantial environmental and health hazards in its untreated form. It contains elevated concentrations of toxic elements such as lead, arsenic, cadmium, chromium, vanadium, and nickel, which can leach into surrounding soil and groundwater, resulting in environmental contamination (Miricioiu and Niculescu 2020; Chen et al. 2024). Similar leaching concerns have been reported in major coal-producing countries, where ash disposal in unlined ponds or open dumps leads to the migration of metals into water resources, threatening drinking water security (Jahandari et al. 2023). Furthermore, the fine particles of CFA, often laden with these hazardous metals, are easily dispersed by wind, contributing to airborne pollution (Tumane et al. 2022; Jahandari et al. 2023). Inhalation of these particulates can lead to serious health issues, including respiratory illnesses, cardiovascular diseases, compromised immune function, and increased risk of lung cancer, particularly among power plant workers and nearby communities (Chen et al. 2024). These health burdens mirror patterns observed in other industrial pollution settings, underscoring the need for systematic risk management and valorization strategies to minimize exposure pathways and minimize their ecological footprint.

### Abundance of SiO₂ and Al₂O₃ in CFA and their value

CFA’s amorphous phase is predominantly composed of silica (SiO₂) and alumina (Al₂O₃). These occur mainly as aluminosilicate minerals, accompanied by smaller amounts of oxides such as Fe₂O₃, CaO, and TiO₂, as well as crystalline phases like quartz and mullite (van der Merwe et al. 2017). The data presented in Table 1 indicate that the majority of CFA samples worldwide contain more than 60 wt% of SiO₂ and Al₂O₃ combined, highlighting its potential as a standardized and abundant feedstock for high-value material recovery. While this compositional consistency is promising, variations in minor oxides (e.g., Fe₂O₃, CaO, TiO₂) and class type (Class F vs. Class C) can significantly influence extraction efficiency and the choice of processing methods.

**Table 1:** Reported Al₂O₃ and SiO₂ composition of CFA and their corresponding source locations and class types

Study no	Al₂O₃ (%)	SiO₂ (%)	References	Ash source/location	Class type
1	27.8	55.9	Ahmad et al. (2020)	Manjung Power Station, Lumut, Perak, Malaysia	Class F
2	29.8	55.6	Das and Rout (2019)	National Thermal Power Corporation, Bongaigaon, Assam, India	Class F
3	40.3	48.7	Han et al. (2020)	CHN Energy, Shanxi, China	Class F
4	32.7	52.6	Imoisili and Jen (2022)	South African thermal power plant	Class F
5	33.7	51.6	Imoisili and Jen (2024)	Komati Power Station, Mpumalanga province, South Africa	Class F
6a	19.6	46.9	Jiang et al. (2020)	Kingston Plant, Tennessee, USA	Class F
6b	30.0	42.8	Jiang et al. (2020)	Electric Power Fly Ash Co. Ltd., Shanghai, China	Class C
7	24.6	49.7	Koshlak (2023)	Polish thermal power plant	Class F
8	38.4	45.5	Koshy et al. (2019)	Zhangjiakou Power Plant, Hebei, China	Class F^1^
9	21.2	53.4	Lei et al. (2024)	Coal-burning power station at Money Point, Clare, Ireland	Class F
10	28.2	64.2	Mudgal et al. (2019)	Satpura Thermal Power Plant, Sarni, Madhya Pradesh, India	Class F
11	22.0	63.0	Naveed et al. (2019)	Lakhra Power Plant, Sindh, Pakistan	Class F^1^
12	20.1	55.6	Nikolić et al. (2016)	Source not reported	Class F^1^
13	32.3	52.8	Pan et al. (2023)	Guang’an Power Station	Class F^1^
14	36.4	50.7	Rożek et al. (2018)	Polish Power Plant	Class F^1^
15	28.5	57.4	Samantasinghar and Singh (2018)	Captive power plant, Rourkela Steel Plant, Odisha, India	Class F
16	38.2	49.6	Wang et al. (2024)	Pingshuo Fired Power Plant, Shanxi, China	Class F^1^

Class F^1^ estimated based on oxide composition (SiO₂ + Al₂O₃ + Fe₂O₃ ≥ 70%) according to ASTM C618/AASHTO M295, as not reported in the original study

Multiple studies have demonstrated the feasibility of recovering these oxides (SiO₂ and Al₂O₃), though reported efficiencies vary due to differences in extraction techniques, ash source, and treatment conditions. For example, Zhao et al. (2023) employed a cascade extraction process, achieving 66% SiO₂ and 90% Al₂O₃ recovery, whereas Xing et al. (2024) reported considerably lower efficiencies (~ 41–42%) using mild hydrothermal processing. In another study, Wang et al. (2024) achieved very high recoveries (~ 87–89%) through low-temperature alkaline leaching combined with Bayer digestion, indicating that optimized chemical conditions can significantly enhance yields. Pressurized sulfuric acid leaching, as applied by Wang et al. (2021), reached 83% aluminum extraction, showing that strong acids remain competitive for alumina recovery despite potential environmental challenges. Among the discussed methods, cascade leaching systems outperform mild hydrothermal routes, mainly due to their ability to disrupt stable aluminosilicate networks and enhance solubilization of reactive phases. However, high-efficiency methods often involve increased chemical consumption, energy input, or environmental considerations, highlighting a trade-off between recovery efficiency and process sustainability. Ultimately, extraction performance depends on both method and material, as shown by multiple recent studies (Collins et al. 2023; Koshlak 2023; Li et al. 2024; Lei et al. 2024; Murmu et al. 2024; Rosita et al. 2024; Widowati et al. 2024; Xing et al. 2024), which highlight that CFA’s variable composition precludes a universal treatment strategy.

In addition to CFA, various agricultural and industrial wastes have been investigated as alternative sources of alumina and silica for sustainable material recovery (Table 2). These wastes differ in oxide composition, mineral reactivity, production scale, and the extent to which they have been successfully valorized, all of which affect their technological feasibility. For example, rice husk, a widely available agricultural byproduct in rice-growing regions, produces rice husk ash upon combustion, which is remarkably rich in silica, containing approximately 87–97% SiO₂ (Nzereogu et al. 2023). This material has been widely used for synthesizing silica gel and nanoparticles (Nzereogu et al. 2023; Hamidu et al. 2025); Islam et al. 2024a, b). However, its high dependency on combustion conditions and limited geographic availability constrain its large-scale adoption (Hamidu et al. 2025); Islam et al. 2024a, b). In contrast, red mud, a byproduct of alumina extraction from bauxite ore, contains approximately 10–89% Al₂O₃ and 3–13% SiO₂ (Vasyunina et al. 2020; Silveira et al. 2021). Despite its high alumina content, much of the aluminum is bound in thermodynamically stable mineral phases, requiring energy-intensive activation or acid leaching for extraction (Duraisamy and Chaunsali 2025). This challenge, coupled with its high alkalinity, continues to hinder full-scale valorization, and its production volumes are relatively low compared to other waste streams like CFA. Another industrial byproduct, like blast furnace slag, contains only moderate levels of silica (30–40% SiO₂) and alumina (10–15% Al₂O₃) (Sajid et al. 2019), which are considerably lower than those found in CFA. While valuable, this material is already heavily recycled in the cement industry across Europe, North America, and parts of Asia, including Japan and Singapore, leaving limited scope for novel aluminosilicate recovery pathways (Kumar et al. 2018; Tole et al. 2020; FM Metal 2024). Overall, these trends reveal that oxide content alone is not a sufficient indicator of recovery potential; rather, the interplay between composition, phase reactivity, and availability dictates actual feasibility. In this context, CFA remains uniquely positioned, combining a favorable Si/Al ratio, amorphous structure, abundant global supply, and compatibility with both alkaline and acid extraction systems. Given these favorable characteristics, CFA represents a promising precursor for high-purity alumina and silica production materials that are central to emerging technologies and circular economy initiatives.

**Table 2:** Sources of alumina and silica for sustainable material recovery

Waste source	Al_2_O_3_ and SiO₂ content (%)	Common valorization products	Critical remarks/research insights	Source
Coal fly ash	Al₂O₃: 20–55; SiO₂: > 60	Zeolites, sodium silicate, alumina, nanoparticles	Among all residues, CFA offers the most balanced Si/Al ratio and amorphous reactivity, making it a model feedstock for aluminosilicate synthesis. However, variability across coal types and trace heavy metals complicates standardization and large-scale reuse, highlighting the need for compositional classification frameworks	Zhang et al. (2021); Aphane et al. (2020); Miricioiu and Niculescu (2020); Chen et al. (2023); Zhou et al. (2022); Aphane et al. (2024); Koshlak (2023); Xing et al. (2024); Xu et al. (2025)
Rice husk ash	SiO₂: 85–95	Silica gel, silica nanoparticles	Its ultra-high silica content makes RHA a benchmark for low-cost, high-purity silica extraction. Yet, inconsistent combustion control often yields crystalline silica, reducing solubility and reactivity. Emerging work on controlled pyrolysis and sol–gel integration aims to overcome this bottleneck	Malpani and Goyal (2023); Nzereogu et al. (2023); Islam et al. (2024a, b); Taiye et al. (2024); Hamidu et al. (2025)
Red mud (bauxite residue)	Al₂O₃: 10–89; SiO₂: 3–13	Alumina, adsorbents	The most alumina-rich but also the most problematic residue—its high alkalinity, fine texture, and heavy metal content impede direct reuse. The research frontier is shifting toward simultaneous neutralization and resource recovery to turn red mud from liability to raw material	Vasyunina et al. (2020); Silveira et al. (2021); Duraisamy and Chaunsali (2025)
Slag (blast furnace)	Al₂O₃: 10–21, SiO₂: 30–40	Geopolymers, cement production, construction material, and soil improvement	Chemically stable with moderate reactivity; its utility lies in high-volume applications. Research trends emphasize hybrid alkali–lime activation systems and CO₂ sequestration routes to enhance circularity in the metallurgical sector	Kumar et al. (2018); Sajid et al. (2019); Tole et al. (2020); FM Metal (2024)
Sugarcane bagasse ash	Al₂O₃: 4.7–9.1; SiO₂: 60–70	Silica, adsorbents, concrete, and soil improvement	SBA bridges agricultural and industrial valorization—moderate silica levels support blended cement and composite adsorbents. Challenges remain in controlling unburnt carbon and ash fineness to achieve consistent pozzolanic activity calcination	Chindaprasirt and Rattanasak (2020); Singh et al. (2021); Thomas et al. (2021); Singh et al. (2022); Zachariah and Jakka (2022)
Oil palm ash	SiO₂: 55–60	Silica, adsorbents, and concrete	Underexploited in the circular economy. Though compositionally similar to SBA, OPA is often land-disposed due to poor collection and classification systems. Future work should emphasize decentralized valorization schemes in oil-producing regions	Wahab et al. (2019); Bukit et al. (2022); Kuswa et al. (2024)

High-purity silica and alumina have attracted increasing interest due to their diverse applications in catalysis, energy storage, membranes, environmental remediation, sensors, and advanced materials manufacturing. Recovering these materials from CFA not only diverts large volumes of waste from ash dumps but also supports a circular economy model by converting an environmental liability into a sustainable feedstock for high-value nanomaterials. This aligns with global trends toward green manufacturing and resource recovery. Despite this potential, existing studies often focus on individual extraction techniques without systematically linking them to process efficiency or environmental considerations. This review fills that gap by providing a comprehensive, critical, and comparative analysis of extraction methods, evaluating their scalability, sustainability, and suitability for producing high-purity precursors for nanomaterials. Specifically, this review aims to:

- Critically assess and categorize existing silica and alumina extraction technologies from CFA
- Compare their efficiency, scalability, and environmental implications
- Highlight novel approaches for converting recovered materials into nanoparticles
- Identify key knowledge gaps and future research directions to advance CFA valorization

By addressing these aspects, this review offers a novel synthesis of the current state of knowledge and a roadmap for advancing CFA valorization toward high-value applications.

## Extraction methods for Al and Si content from coal fly ash

This section reviews extraction methods for aluminum (Al) and silicon (Si) from coal fly ash (CFA). Both elemental and their oxides data are presented to capture the full scope of reported results and enable meaningful comparisons across studies.

### Sintering processes

Traditionally, aluminum recovery has dominated the literature, largely because alumina-bearing phases in CFA, such as amorphous aluminosilicates, offer high economic value and are often more accessible. In contrast, silicon extraction has received less focus historically, as silicon is typically locked within more stable silicate structures, making it harder to extract (Yao et al. 2014; Aphane et al. 2015). Conventional aluminum recovery from CFA and other industrial wastes like red mud has relied heavily on sintering-based methods (Chen et al. 2023a, b). These include the lime sinter process, lime–soda sinter process, lime-soda-potassium carbonate combinations, the Calsinter process, and various sinter modifications (e.g., salt-soda sinter, ammonium sulfate sinter, self-disintegrating sinter, and fluorides sinter), as summarized in Table 3. Sintering involves blending the waste material with chemical additives and heating the mixture to high temperatures (typically 800–1400 °C) to trigger solid-state reactions that convert inert mineral phases into soluble compounds (ElDeeb et al. 2020). The sintering techniques have proven effective in breaking down refractory phases like mullite and improving aluminum leachability (Pereira et al. 2014).

**Table 3:** Sinter-based processes for aluminum extraction from CFA

Process category	Technology	Advantages	Disadvantages	Lime requirement and environmental impact	References
Lime-only sinter processes	Lime sinter process	Auto-disintegration eliminates grinding; lime is inexpensive	Generates considerable calcium silicate residues which are often inert and unsuitable for further chemical recovery	High lime consumption; environmental hazard due to calcium silicate waste. Most recent studies post-2020 focus on hybrid activation, with very few works on pure lime sintering	Hignett (1947); Chou et al. (1975); Padilla & Sohn (1985b); Eriksson & Björkman (2004); Guzzon et al. (2007); Lin et al. (2012); Yao et al. (2014)
	Calsinter process	Cost-effective; dissolves aluminum and other metals with dilute acid	Soluble impurities may contaminate the leach solution; the process is complex	Low lime requirement; acid leaching may pose contamination risk	Goodboy (1976); Egan et al. (1979); Seeley et al. (1981); Egan et al. (1981); Kelmers et al. (1982)
Lime–soda sinter processes	Lime–soda sinter process	Produces fewer residues than lime-only sinter; improved alumina extraction	High energy consumption; complex process; soda is costly	Moderate lime use; fewer residues than lime-only sinter; still poses environmental concerns	Padilla & Sohn ( 1985b); Kayser (1902); Bai et al. (2010); Wang et al. (2012); Tian et al. (2016); Beranova et al. (2019); Bakirov et al. (2024)
	Predesilication + lime–soda sinter	Enables simultaneous silica extraction; reduces solid residues	Complex process; filtration and washing challenges	Moderate lime use; reduced waste compared to individual sintering	Wang et al. (2012); Jiang et al. (2015)
Hybrid/mixed sinter processes	Other sinter processes (salt–soda, ammonium sulfate, self-disintegrating, fluorides)	Varied methods allow flexibility and optimization; potential for lower residue	Often complex; some methods use hazardous reagents; limited performance data	Lime use varies; some processes use alternative fluxes; environmental risk depends on reagents	Decarlo et al. (1978); McDowell & Seeley (1980); Nehari et al. (1999); Park et al. (2004); Tong et al. (2008); Bian et al. (2025)
	Sinter–acid leach/activation-assisted	High extraction efficiency (up to 97% Al); converts mullite into acid-soluble forms; suitable for post-2020 studies	Complex and energy-intensive; handling strong acids	May require lime in the sinter phase; hybrid process improves extraction but increases chemical input	Bai et al. (2011); Liu et al. (2012); Shemi et al. (2015); Liu et al. (2016); Bian et al. (2025); Zhao et al. (2025)

The lime-sinter process, derived from the Pederson method, recovers alumina from CFA by reacting it with lime (CaO) at elevated temperatures, forming soluble calcium aluminates and insoluble calcium silicates (Yao et al. 2014). Upon cooling, the sintered material auto-disintegrates into fine powder, facilitating the separation of aluminum from silicon during leaching with alkaline solutions. Aluminum in the leachate is precipitated as Al(OH)₃ and subsequently calcined to yield high-purity α-Al₂O₃, while the remaining silicate residues are retained (Yao et al. 2014). The advantage of this method lies in relatively simple chemistry and the ability to recover high-purity alumina. Lime-only sintering is energy-intensive and consumes large amounts of CaO (CaO/Al₂O₃ > 1.0). Furthermore, alumina leaching is limited due to the formation of stable calcium aluminates. The process also produces large slag volumes, which complicate residue handling and reduce efficiency. The Calsinter method was then developed to overcome these limitations. It uses lower amounts of CaSO₄ and CaCO₃ with CFA. This forms aluminates that are easier to leach with acid, reduces slag formation, and improves alumina recovery. As evidence of the effectiveness of this method, laboratory tests by Kelmers et al. (1982) reported aluminum solubilization of 95–98%, though the method requires acid treatment and careful waste management.

The lime–soda sinter process, originally developed by Kayser in 1902 for bauxite, was later adapted for CFA aluminum extraction. In this method, CFA is reacted with a mixture of CaO and soda (Na₂CO₃ or NaOH) at high temperatures, forming soluble sodium calcium aluminates and insoluble calcium silicates (Yao et al. 2014). The sintered product is leached with alkaline solutions, silicon is removed using Ca(OH)₂, and aluminum is precipitated as Al(OH)₃ before calcination. Adaptations of this process, such as those described by Padilla and Sohn (1985a) and Yao et al. (2014), reported alumina recoveries of up to ~ 80% under optimized conditions. To enhance the efficiency of the lime–soda sinter process, predesilication can be introduced as a preparatory step. This approach enriches the Al₂O₃/SiO₂ ratio, reduces the required amount of sintering agents, and promotes the formation of more reactive alumina phases and increases alumina recovery above 90%, while the removed silica can be valorized (Wang et al. 2012; Bai et al. 2010). Bakirov et al. (2024) reported that sintering ash and slag waste using a lime–soda process achieved up to 90.2% alumina extraction, compared with ≤ 60% for lime sinter alone, demonstrating how soda enhances recovery and produces self-disintegrating sinters suitable for leaching.

Recent advances focus on hybrid or mixed sinter processes, combining high-temperature activation with chemical treatments to improve extraction efficiency from highly polymerized matrices like mullite and sodium aluminosilicate. For example, synergistic potassium pyrosulfate–ammonium sulfate roasting–leaching achieved 94.95% alumina extraction by selectively activating mullite (Bian et al. 2025), while sodium carbonate flux activation followed by hydrochloric acid leaching reached up to 97% extraction (Zhao et al. 2025). These methods demonstrate improved efficiency, selective aluminum recovery, and potential reduction of lime usage, but they remain more complex, energy-intensive, and require careful handling of strong acids or fluxes.

While classical sintering approaches remain important for understanding aluminum mobilization in CFA, their limitations underscore the need for complementary strategies. Recent mixed-sinter methods selectively activate aluminum-bearing phases without fully relying on high temperatures, creating opportunities to combine chemical activation with targeted extraction techniques. These findings provide a clear rationale for exploring acid-based extraction methods, which can build on the selective phase conversion initiated by sintering while reducing environmental impact and operational demands.

### Direct acid leaching

Direct acid leaching is one of the most extensively studied methods for aluminum recovery from CFA, owing to its ability to selectively dissolve aluminum while leaving silica largely intact due to its chemical stability in acidic media (Yao et al. 2014; Aphane et al. 2015). In this process, aluminum is leached into solution, while silicon remains in the solid residue, allowing for efficient separation (Aphane et al. 2020). The resulting aluminum-rich leachate can be further processed into high-value products, while the silicon-rich residue offers potential for conversion into silica-based materials (Aphane et al. 2020). This dual recovery route significantly improves the sustainability and economic viability of CFA valorization. The leaching procedure typically involves treating CFA with strong acids such as hydrochloric acid (HCl), sulfuric acid (H₂SO₄), or nitric acid (HNO₃) at elevated temperatures (Wu et al. 2012; Shemi 2013; Yao et al. 2014; Sangita and Panda 2016). Under these conditions, amorphous and partially crystalline aluminum phases dissolve readily, releasing Al^3^⁺ ions into solution. However, aluminum embedded in mullite, a highly stable and thermodynamically resilient mineral phase in CFA, exhibits low solubility in acid, rendering it largely resistant to this treatment (Shemi 2013; Cui et al. 2024). Therefore, the direct acid leaching is particularly effective for extracting aluminum from the amorphous fractions of CFA rather than from crystalline aluminosilicates.

The leaching of alumina typically proceeds through well-known acid–base reactions, converting solid Al₂O₃ into soluble aluminum salts. The reactions in Eqs. (1), (2), and (3) illustrate the dissolution of alumina into an aluminum-rich leachate (Lisbona et al. 2013; Shi et al. 2024). Aluminum can then be recovered from the leachate through various processes, including neutralization with alkaline agents such as sodium hydroxide (NaOH) or ammonium hydroxide (NH₄OH), which precipitates aluminum hydroxide [Al(OH)₃] (Mwase et al. 2024; Shi et al. 2024). Other methods include electrolytic smelting, electrochemical precipitation, solvent extraction, CO₂-induced precipitation, and ion exchange, each offering different advantages for selective aluminum recovery (Shemi 2013; Lysenko and Kondrat’eva 2020; Wang et al. 2020; Shi et al. 2024; Fakhrurozi et al. 2025). The precipitated Al(OH)₃ can then be calcined to produce high-purity alumina (Al₂O₃) for various applications (Fakhrurozi et al. 2025).

1 $$Al_2O_3\:+\:6HCl\:\rightarrow\:2AlCl_3\:+\:3H_2O$$

2 $$Al_2O_3\:+\:3H_2SO_4\:\rightarrow\:Al_2{(SO_4)}_3\:+\:3H_2O$$

3 $$Al_2O_3\:+\:6HNO_3\:\rightarrow\:2Al{(NO_3)}_3\:+\:3H_2O$$

Several studies have provided evidence supporting and optimizing the use of direct acid leaching for aluminum recovery from CFA. For example, Sangita and Panda (2016) reported a maximum extraction efficiency of 69% using sulfuric acid at 220 °C with a 1:3 solid-to-liquid ratio. Their findings also highlighted that increasing acid concentration and adjusting the solid-to-liquid ratio significantly improved extraction performance. Aphane et al. (2015) achieved up to 80% alumina recovery from the amorphous glass phase of CFA using 5 M H₂SO₄ under reflux at 95 °C for 4 h. This study also noted the co-dissolution of silica and alumina under milder acid conditions, suggesting that process selectivity depends strongly on acid strength and operating temperature. In another investigation, Li et al. (2011) reported an aluminum recovery of up to 87% under optimized conditions of 200–210 °C, 80 min residence time, and a 5:1 volumetric acid-to-ash ratio. Elsewhere, Thamilselvi and Balamurugan (2018) demonstrated that treating CFA with 6 M H₂SO₄ at 60 °C for 240 min (S:L ratio 1:5) achieved a high alumina recovery of 94%, highlighting the effectiveness of direct acid leaching under moderate conditions. Collectively, these findings show direct acid leaching as a technically mature and tunable method capable of high recovery yields under optimized conditions, making it a competitive alternative or complement to sintering-based processes.

#### Effect of acid concentrations on extraction

Acid concentration is one of the key factors influencing aluminum extraction efficiency in the direct acid leaching process of CFA. Stronger acids enhance proton availability, promoting the dissolution of alumina phases and increasing extraction yields (Aphane et al. 2015). However, excessively high acid strengths can trigger side reactions such as secondary precipitate formation or passivation layers that impede leaching (Shemi 2013). Several studies have investigated this effect under varying conditions. Sangita and Panda (2016) evaluated multiple acids (HCl, HNO₃, H₃PO₄, and H₂SO₄) at different concentrations (normality) and temperatures. The highest aluminum recovery (42%) was obtained using H₂SO₄ at 220 °C with concentrations increasing from 4 to 41.1 N. HCl (2–11.3 N) at 110 °C, and HNO₃ (2–11.3 N) at 120 °C yielded lower recoveries of 6% and 4%, respectively. Consistent with these results, Yao et al. (2014) emphasized that mild conditions, such as low acid concentrations or ambient temperature, are generally ineffective for high-yield aluminum extraction. Similarly, Shemi et al. (2014) applied the direct acid leaching method using H₂SO₄ and achieved only 24% aluminum recovery, highlighting the limitations of insufficient acid strength. In another study, Nayak and Panda (2010) also confirmed that low acid concentrations at ambient temperatures are inadequate for effective aluminum extraction from CFA. A common issue with acid leaching, especially at low concentrations, is the co-dissolution of silica, which can form gelatinous aluminum–silica residues that hinder filtration and reduce overall recovery (Verbaan and Louw 1989; Aphane et al. 2015).

Further insights into the relationship between acid concentration (molarity) and alumina recovery were provided by Aphane et al. (2015), who observed that increasing H₂SO₄ concentration from 3 to 5 M significantly enhanced alumina extraction to 80%. This improvement was linked to greater availability of hydronium ions, which accelerates the leaching reactions. However, the extraction efficiency declined beyond 5 M (e.g., at 8–10 M). The decrease was attributed to the formation of aluminum sulfate precipitates on the CFA surface, which blocked acid access to unreacted aluminum and hindered further dissolution. In a more detailed mechanistic study, Wu et al. (2012) investigated the effect of sulfuric acid concentration ranging from 30 to 60%. Aluminum extraction efficiency increased from 66 to 82% as acid concentration rose to 50%, due to more effective dissolution of alumina-containing minerals such as mullite. In this study, the X-ray diffraction (XRD) analysis confirmed the breakdown of mullite, with concurrent increases in quartz and calcium sulfate phases. However, at 60% H₂SO₄, recovery unexpectedly dropped to 58%. This decline was attributed to forming a viscous layer composed primarily of aluminum sulfate pentahydrate, calcium sulfate, residual mullite, and quartz. This gelatinous layer inhibited aluminum diffusion into the bulk solution and reduced mass transfer, thereby limiting extraction despite continued mineral dissolution.

These findings underscore that while increasing acid concentration generally enhances recovery, excessively high concentrations may cause secondary reactions and diffusion limitations that offset the benefits, indicating the need for careful optimization of leaching parameters.

#### Effect of reaction time on extraction

The duration of acid leaching significantly influences aluminum/alumina recovery from CFA. Longer reaction times typically enhance contact between the acid and reactive aluminum-bearing phases, improving extraction efficiency. However, excessively prolonged leaching can lead to diminishing returns or even reduced recovery due to secondary effects such as re-precipitation of dissolved species or formation of interfering residues. As evidence, Wu et al. (2012) demonstrated that prolonging reaction time significantly enhanced aluminum recovery, with extraction efficiency rising from 33 to 86% as the leaching time increased from 60 to 300 min. This was attributed to the slow dissolution of aluminum–silicon glass phases in the CFA, which required extended contact time with the acid to achieve complete decomposition. In another study, Aphane et al. (2015) systematically studied the effect of leaching duration on alumina extraction using sulfuric acid at varying concentrations. At a low acid concentration of 1 M H₂SO₄, alumina recovery showed a minimal increase from 36% at 30 min to 39% at 240 min, due to limited proton availability. In contrast, at the optimal concentration of 5 M H₂SO₄, recovery rose sharply, reaching 80% after 240 min. However, extending the leaching time to 350 and 500 min led to a steep decline in recovery below 50%. This drop was attributed to forming a passivating aluminum sulfate layer on CFA particle surfaces, which restricted acid access to unreacted aluminum. Additionally, excess sulfuric acid promoted the precipitation of aluminum sulfate from the leachate, further reducing alumina recovery (Aphane et al. 2015). Similar findings were reported by Bai et al. (2011), who observed that aluminum extraction efficiency increased from 5% at 30 min to 80% at 120 min. However, a decline in efficiency was noted with longer leaching durations, likely due to precipitation of aluminum sulfate and silica complexes at elevated temperatures. Collectively, these studies highlight that while increased reaction time can improve the recovery, there is an optimal duration beyond which efficiency declines due to secondary reactions and physical barriers limiting further extraction. These observations also suggest that excessively long durations add energy costs; therefore, defining an optimal leaching time is key to improving process feasibility.

#### Effect of temperature

Temperature plays a critical role in enhancing extraction efficiency during acid leaching of CFA. Elevated temperatures accelerate reaction kinetics and promote the breakdown of thermally stable alumina-containing minerals. Wu et al. (2012) markedly improved aluminum recovery, increasing from 50% at 140 °C to 86% at 220 °C. This improvement was attributed to the thermal decomposition of refractory phases such as mullite and aluminum–silicon glass, which begin to degrade above 150 °C. Infrared spectral analysis confirmed this transformation, showing diminished mullite-related absorption bands and increased quartz signatures after leaching at elevated temperatures (140–180 °C), indicating the breakdown of aluminosilicate phases and quartz enrichment. Supporting this, Bai et al. (2011) reported minimal aluminum recovery below 100 °C, with significant CFA decomposition and peak extraction observed at 275 °C. Harada et al.’s (1993) findings also aligned, showing substantial mullite degradation at 230 °C after 16 h of leaching. Collectively, these studies underscore the importance of high temperatures in disrupting stable mineral matrices, thereby enhancing aluminum release during acid leaching. It is imperative to note that elevated temperatures also increase energy demand and may influence effluent composition. Therefore, implementation of this method requires thorough temperature optimization to maximize dissolution without disproportionately increasing operational costs.

#### Influence of solid-to-liquid ratio and particle size

The extraction from CFA is significantly influenced not only by chemical and thermal conditions but also by physical factors such as the solid-to-liquid (S:L) ratio and particle size. These parameters govern the contact between the leaching agent and CFA particles, directly impacting leaching efficiency. Sangita and Panda (2016) observed that increasing the S:L ratio from 1:1 to 1:4 using sulfuric acid enhanced aluminum recovery from 42 to 69%, attributed to greater acid availability and improved mass transfer. Similarly, Bai et al. (2011) reported an increase in aluminum extraction from 60% at a 1:0.8 ratio to 90% at 1:1.4. Particle size also plays a key role. For example, Wu et al. (2012) found that reducing the particle size from 243 to 74 μm improved aluminum recovery, likely due to the increased reactive surface area. The authors further noted that the extraction reduced below 74 μm, possibly due to particle agglomeration limiting further surface accessibility. In another study, Bai et al. (2011) similarly showed that decreasing particle size from 160 to 10 μm raised aluminum recovery from around 40% to over 85%. These observations highlight that beyond chemical and thermal optimization, careful control of physical parameters is crucial for efficient aluminum recovery from CFA.

#### Synthesis

Among the acids investigated, sulfuric acid (H₂SO₄) has been the most widely adopted and consistently yields the highest aluminum extraction efficiencies (up to 94%), owing to its strong acidity, low cost, and process stability (Table 4). In contrast, phosphoric acid (H₃PO₄) has shown considerably lower extraction performance (41%), while hydrochloric acid (HCl) and nitric acid (HNO₃) have been found to be largely ineffective (Sangita & Panda 2016).

**Table 4:** Alumina recovery efficiencies from CFA using direct acid leaching methods

Reagent type	Acid concentration (M)	Optimal conditions	Efficiency (%)	References
H₂SO₄	5.0	210 °C; 80 min; S:L = 1:5	Al—87	Li et al. (2011)
H₃SO₄	6.0	75 °C; 360 min; S:L = 1:4	Al—25	Shemi (2013)
H₂SO₄	5.0	95 °C; 240 min; S:L = 1:10	Al₂O₃—80	Aphane et al. (2015)
H₂SO₄	18	220 °C; 240 min; S:L = 1:3	Al—69	Sangita and Panda (2016)
H₃PO₄	13.7	220 °C; 240 min; S:L = 1:3	Al—41	Sangita and Panda (2016)
H₂SO₄	6.0	60 °C, 240 min; S:L 1:5	Al₂O₃—94	Thamilselvi & Balamurugan (2018)

Overall, this method offers high extraction efficiency for amorphous phases with lower energy requirements than sintering-based methods. Its operational simplicity and flexibility make it technically viable for industrial deployment. Unlike sintering, direct acid leaching minimizes residue generation but requires careful management of acidic effluents, introducing a different set of environmental trade-offs. Although direct acid leaching is mature for alumina/aluminum recovery, its effectiveness remains limited by the chemical stability of mullite, which restricts full resource utilization from CFA. This highlights a clear research gap, which requires improved pre-activation strategies or synergistic combinations with other treatments to enhance aluminum liberation from crystalline phases. Therefore, future work should focus on integrated process design that combines this method with activation (like sintering process) or selective separation steps while emphasizing effluent recovery and circular chemical use to improve environmental performance. Categorically, direct acid leaching represents a transition from thermally intensive to chemically selective valorization strategies, marking an important evolution in CFA processing.

### Enhanced acid leaching process

Although conventional direct acid leaching has proven effective for extracting aluminum from CFA, advanced methods incorporating pre-treatment and process intensification techniques have been developed to substantially boost extraction efficiency. Enhanced acid leaching techniques are designed to address challenges associated with the mineralogical complexity and low reactivity of CFA. These approaches can be systematically grouped into pressure-assisted leaching, sinter-acid leaching, lime-assisted calcination, and microwave-assisted activation methods. Each category targets different reactivity barriers, either by enhancing mineral breakdown kinetics, increasing surface reactivity, or optimizing dissolution selectivity.

Pressure acid leaching utilizes elevated temperatures and pressures to accelerate the breakdown of alumina-containing phases. For example, Wu et al. (2012) applied this method by treating CFA having a particle size of 74 with 50% sulfuric acid at 180 °C for 4 h, achieving an aluminum extraction efficiency of 82%. Sinter-acid leaching is another effective strategy that integrates thermal activation with subsequent acid treatment. This approach minimizes acid consumption and avoids the use of fluorine-based additives, as they are potentially hazardous to the environment. Studies by Bai et al. (2011) and Liu et al. (2016) showed that thermally treating CFA with concentrated sulfuric acid converts alumina into water-soluble aluminum sulfate, which can then be efficiently extracted using hot water. These methods achieved alumina recovery rates of 70–90% under relatively mild conditions while also generating minimal solid residue. Another enhanced acid leaching method is lime-assisted calcination, investigated by Matjie et al. (2005), in which CFA was treated with CaO at 1000–1200 °C to form reactive calcium aluminate phases. Subsequent leaching with 6 mol/L sulfuric acid for 4 h achieved an alumina extraction efficiency of 85%. Li et al. (2009) extended acid leaching applications to boiler slag, achieving 87% aluminum extraction using 4 mol/L H₂SO₄ at 80 °C for 24 h with a solid-to-liquid ratio of 1:5. Additionally, microwave-assisted pre-treatment has emerged as a promising innovation. Ma et al. (2021) developed a microwave-assisted baking and leaching method for the simultaneous recovery of aluminum and titanium. Under optimized conditions (baking at 280 °C for 60 min with 1.2 times the stoichiometric reagent amount, followed by leaching at 60 °C for 30 min with a liquid-to-solid ratio of 5:1), extraction efficiencies reached 82% for aluminum and 56% for titanium.

From a performance perspective, enhanced acid leaching of CFA demonstrates that careful optimization of reagents, temperature, and reaction time can significantly improve recovery (Table 5). These results also highlight how operating conditions, choice of reagents, and pre-treatment strategies collectively influence both recovery efficiency and process sustainability, illustrating the clear advantages of enhanced acid leaching over conventional acid leaching.

**Table 5:** Alumina recovery efficiencies from CFA using enhanced acid leaching methods

Extraction system	Acid concentration (M)	Optimal conditions	Efficiency (%)	References
H₂SO₄ in a pressure reaction kettle	~ 9.0^a^	180 °C; 240 min	Al—82	Wu et al. (2012)
Calcination with H₂SO₄	16–17^a^	275 °C; 120 min; S:L = 1:1.4	Al₂O₃—85	Bai et al. (2011)
Sintering–H₂SO₄ process	~ 18^a^	200 °C; 240 min; S:L = 1:6	Al₂O₃—70–90	Liu et al. (2016)
CaO (lime) and H₂SO₄ system	6.0	CaO at 1000–1200 °C; leaching at 80 °C for 240 min	Al₂O₃—85	Matjie et al. (2005)
Pre-calcination followed by H₂SO₄ leaching	4.0	Pre-calcination at 1000 °C for 120 min; leaching at 80 °C for 1440 min; S/L = 1:5	Al—87	Li et al. (2009)
H₂SO₄–NH₄HSO₄ combined system	Not specified	Baking at 280 °C for 60 min; leaching at 60 °C for 30 min using 1.2 × stoichiometric reagent; S/L = 1:5	Al—82	Ma et al. (2021)

^a^Approximate concentration, converted from reported acid percentage (% w/v)

Enhanced acid leaching of CFA faces significant operational, technical, and environmental limitations. The use of concentrated acids can corrode equipment, generate hazardous waste, and increase costs for handling, recovery, or neutralization. Technically, these methods are inefficient at dissolving silica, which remains largely inert as amorphous or quartz forms, while aluminum-bearing phases such as mullite and aluminosilicate glass are effectively leached (Li et al. 2024; Liu et al. 2024). As a result, high aluminum recovery is achieved at the expense of full resource utilization. Scaling up further introduces challenges, including acid management, material costs, and effluent treatment. Therefore, acid-centered leaching alone is insufficient. Future research should focus on integrating enhanced acid leaching with alkaline or selective separation processes to enable multi-component recovery. Additionally, systematic techno-economic and environmental assessments are needed to guide sustainable and scalable implementation.

### Alkaline leaching

**Fig. 1:**
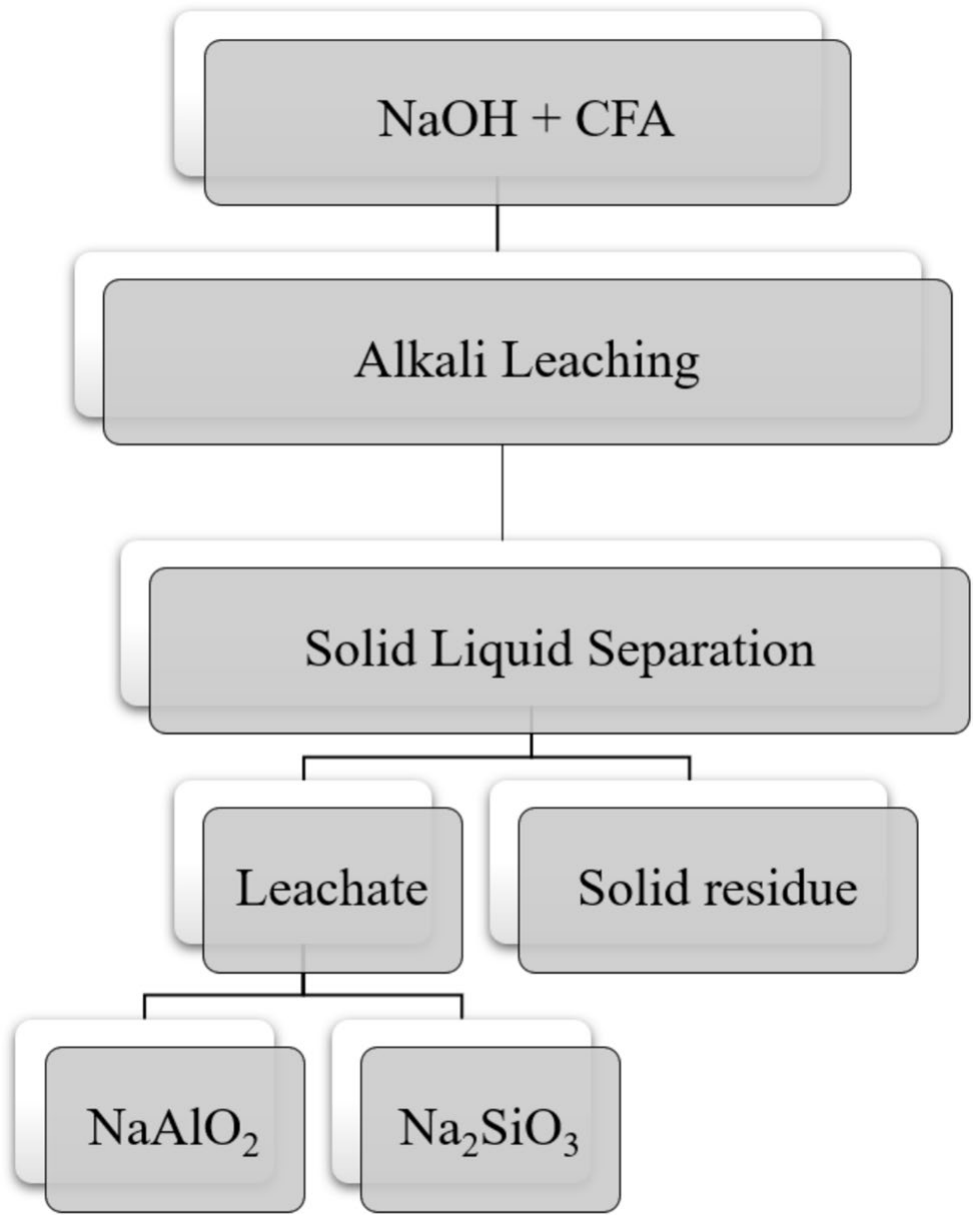
Process flow diagram for the production of sodium silicate and sodium aluminate (Aphane et al. 2024; Murmu et al. 2024)

Unlike acid leaching, alkali leaching selectively dissolves both silica and alumina phases under alkaline conditions, enabling the recovery of these valuable components from CFA. This chemical treatment involves using alkali solutions to break down and solubilize specific mineral phases in the CFA, particularly silica (SiO₂) and alumina (Al₂O₃) (Aphane et al. 2020, 2024; Collins et al. 2023). Sodium hydroxide (NaOH) is the most commonly used alkali in leaching processes due to its high efficiency in dissolving both silica and alumina from CFA (Table 6). Other alkalis, such as potassium hydroxide (KOH) and sodium carbonate (Na₂CO₃), have also been applied, but they tend to be less effective or more expensive (Ju et al. 2023; Liu et al. 2024; Murmu et al. 2024). CFA consists of both crystalline and amorphous aluminosilicate phases (Ma et al. 2021; Valeev et al. 2022). The amorphous phases, which lack a defined crystalline structure, are particularly reactive because of their disordered arrangement and higher surface energy compared to crystalline minerals like quartz (Lei et al. 2024). These amorphous aluminosilicates are key contributors to the alkali leaching process. When exposed to an alkali solution, they break down and release soluble aluminate (NaAlO₂) and silicate (Na₂SiO₃) species (Murmu et al. 2024), as shown by Fig. 1. This dissolution facilitates the extraction and recovery of alumina and silica from the CFA. The underlying reaction mechanism involves the disruption of the aluminosilicate network, yielding soluble products as illustrated in the equation below:

**Table 6:** Performance comparison of alkaline leaching methods processes for silica and alumina recovery from CFA

Reagent type	Alkaline concentration (M)	Optimal conditions	Efficiency (%)	References
NaOH	10	95 °C; 60 min; S:L = 1:10	SiO₂—60; Al₂O₃—17	Aphane et al. (2020)
NaOH	5.0^a^	120 °C; 120 min; S:L = 1:5	SiO₂—34; Al₂O₃—1.4	Xing et al. (2024)
NaOH	9.5	75 °C; 90 min; S:L = 1:10	Si—71; Al—72	Widowati et al. (2024)
NaOH	10	65 °C; 90 min; S:L = 1:10	Si—28; Al—32	Rosita et al. (2024)
KOH	6.4^a^	150 °C; 120 min; L:S = 10:1	SiO₂—43	Murmu et al. (2024)
NaOH	6.0^a^	100 °C; 120 min; L:S = 1:5	SiO₂—55	Murmu et al. (2024)

^a^Approximate concentration, converted from reported acid percentage (% w/v)

4 $$Amorphous\;Aluminosilicate\:+\:NaOH\:\rightarrow\:NaAlO_2\:+\:Na_2SiO_3\:+\:H_2O$$

The widely used Bayer process extracts aluminum from the sodium aluminate solution by neutralizing it with water, which precipitates aluminum hydroxide (Al(OH)₃), as shown in Eq. (5) (Gupta and Mukherjee 2019). The aluminum hydroxide is then calcined to form aluminum oxide (Al₂O₃) (Eq. 6), which is further processed to yield metallic aluminum (Eq. 7) (Shemi 2013; Gupta and Mukherjee 2019; Lysenko and Kondrat’eva 2020; Wang et al. 2020; Arnold 2022; Shi et al. 2024; Fakhrurozi et al. 2025).

5 $$NaAlO_2\:+\:H_2O\:\rightarrow\:Al{(OH)}_3\:+\:NaOH$$

6 $$2Al{(OH)}_3\:\rightarrow\:Al_2O_3\:+\:3H_2O$$

7 $$Al_2O_3\:\rightarrow\:2Al\:+\:O_2$$

For silica recovery, the sodium silicate produced from the leaching of amorphous aluminosilicates is treated with an acid, most commonly hydrochloric acid (HCl), which precipitates silica gel (SiO₂·H₂O), as shown in Eq. (8) (Gorrepati et al. 2010; Tognonvi et al. 2011; Rahman et al. 2018; Pan et al. 2023). The silica gel is subsequently dried to yield pure silica (SiO₂), as illustrated in Eq. (9).

8 $$Na_2SiO_3\:+\:2HCl\:\rightarrow\:SiO_2\:+\:2NaCl\:+\:H_2O$$

9 $$SiO_2\cdot H_2O\rightarrow SiO_2+H_2O$$

Given the vast availability of CFA and the necessity for its beneficiation, numerous studies have investigated the extraction of its valuable components. Aphane et al. (2020), for example, successfully recovered sodium silicate from South African CFA using a direct alkaline leaching (DAL) method, which involved a one-step extraction with NaOH. Their results showed substantial silica recovery, 60% from the amorphous phase and 42% overall. Other researchers have reported similar successes using alkaline leaching, with silica recovery ranging from 33% (Xing et al. 2024) to 71% (Widowati et al. 2024), and aluminum recovery ranging from 32% (Rosita et al. 2024) to 72% (Widowati et al. 2024).

#### Effect of alkali concentration

Studies have investigated how varying NaOH concentrations influence the dissolution of key mineral phases. For instance, Collins et al. (2023) found that lower NaOH concentrations, such as 1 M, resulted in only slight mineral dissolution and minimal changes in the mineralogy of CFA. In contrast, higher concentrations, such as 8 M NaOH, led to substantial removal of both SiO₂ and Al₂O₃, indicating a more aggressive breakdown of the ash matrix. This trend is supported by Rosita et al. (2024), who observed better leaching efficiency of both Si and Al with increasing NaOH concentration. However, they also reported a decline in aluminum extraction under certain conditions, potentially due to the formation of secondary mineral phases like katoite and hydroxy sodalite, which tend to immobilize aluminum in the desilicated residue. Furthermore, in the same study, optimal leaching was achieved using 10 M NaOH at 65 °C for 103 min, yielding maximum extraction efficiencies of 28% for Si and 32% for Al. In another study, Widowati et al. (2024) achieved significantly higher extraction efficiencies, 71% for silicon and 72% for aluminum, by employing 9.5 M NaOH at 75 °C, a liquid-to-solid ratio of 10 mL/g, and a reaction time of 90 min in their investigation of rare earth element recovery from high-calcium, iron-rich CFA. Collectively, these results indicate that higher alkali concentrations generally improve the recovery, but it is important to note that this must be balanced against potential increases in operational costs, waste generation, and secondary precipitate formation, highlighting a trade-off between efficiency and sustainability.

#### Effect of temperature

Temperature is another key parameter influencing the alkaline leaching of CFA, as it accelerates reaction kinetics and promotes product formation by shifting equilibrium conditions. Several studies have shown that increases in temperature can enhance the dissolution of SiO₂ and Al₂O₃, primarily due to improved molecular collisions, reduced solution viscosity, and thinner diffusion boundary layers (Xing et al. 2024). As evidence, a silicon-rich solution (SRS) was extracted from CFA using NaOH, achieving a maximum SiO₂ extraction rate of 33% at 120 °C (Xing et al. 2024). However, when the temperature was further increased to 150 °C, the silicon extraction rate dropped sharply to 5%. This decline at higher temperatures was attributed to the formation of secondary phases, particularly hydrated sodium aluminosilicates, that incorporate silicon into stable structures, thereby reducing its solubility and availability in solution. A similar pattern was observed by Su et al. (2011), who found that SiO₂ leaching peaked at 95 °C with a recovery of 38% before declining at higher temperatures. Further evidence from Jiang et al. (2015), Ding et al. (2019), and Ju et al. (2020) supports the notion that excessively high temperatures favor the formation of stable aluminosilicate precipitates, thereby limiting extraction efficiency. In a separate study, Murmu et al. (2024) showed that increasing temperature improved silicon recovery up to 41% using KOH at 150 °C and 51% with NaOH at 100 °C. However, beyond these temperatures, leaching efficiency declined due to the formation of alkali-aluminosilicate phases and zeolitic crystals on the CFA surface, which acted as diffusion barriers to further leaching. This phenomenon was also supported by Faraji et al. (2022), who highlighted that elevated temperatures may ultimately hinder the release of both Si and Al into solution. Overall, temperature enhances kinetics but can induce undesired mineral transformations at extremes, illustrating the importance of precise thermal control for selective leaching.

#### Effect of leaching time

Several studies have demonstrated that silica dissolution tends to increase rapidly during the initial stages of leaching before plateauing over time. For example, Murmu et al. (2024) observed that silica dissolution rose sharply within the first 60 min, achieving over 45% dissolution with KOH at 180 min and 51% with NaOH at 120 min. This trend is largely due to the rapid breakdown of amorphous silica phases in the early reaction stages. Similarly, Rosita et al. (2024) and Widowati et al. (2024) reported optimal extraction efficiencies of 28% and 71% for Si and 32% and 72% for Al, respectively, within a relatively short time (90 min). Xing et al. (2024) further demonstrated that extending the leaching time (up to 4 h) and increasing NaOH concentration (up to 300 g/L) enhanced SiO₂ recovery, reaching 33% and 34%, respectively. However, they also noted diminishing returns beyond 2 h or 200 g/L, as excessive alkali use increased solution viscosity, complicating downstream processes such as dealkalization. These findings indicate that silica and alumina extraction from CFA occurs most rapidly in the early stages of leaching, with higher alkali concentrations and extended times offering limited additional recovery, highlighting the need for optimized conditions to balance efficiency and process manageability.

#### Effect of solid-to-liquid (S/L) ratio

Optimal S/L ratios ensure sufficient dispersion and contact surface area, which promote efficient mass transfer and reaction kinetics. Rosita et al. (2024) identified an S/L ratio of 0.1 g/mL as optimal when used in conjunction with 10 mol/L NaOH at 65 °C for 90 min, achieving digestion efficiencies of 28% for silicon and 32% for aluminum. This finding was corroborated by Widowati et al. (2024), who also reported improved dissolution efficiencies at the same ratio. Murmu et al. (2024) further explored the impact of S/L ratios and noted that at low L/S ratios (e.g., 1:1), the leaching efficiency was poor due to limited mass transfer and particle mobility. Increasing the ratio to 5:1 for NaOH and 10:1 for KOH led to marked improvements, achieving up to 50% and 41% silica dissolution, respectively, owing to better particle suspension and greater surface area exposure. However, beyond these optimal ranges, further increases in dilution led to a decline in leaching efficiency. Murmu et al. (2024) attributed this to the formation and precipitation of alkali silicates, which hindered further dissolution. Similarly, Li et al. (2024) observed that raising the S/L ratio from 1:25 to 1:125 improved leaching of critical elements from 54 to 73%. However, excessive dilution beyond that point reduced efficiency due to a drop in reactant concentration. In contrast, Xing et al. (2024) reported only marginal variation in SiO₂ extraction (29%–32%) when adjusting the L/S ratio from 2 to 10 mL/g, suggesting that in some systems, this parameter may be less influential. From these results, it can be seen that an optimal S/L ratio is a key operational lever influencing both recovery efficiency and downstream processing requirements. Over-dilution can increase reagent use and cost, while under-dilution reduces recovery. Thus, future work should prioritize integrating alkaline leaching with pre-activation steps (e.g., thermal, microwave, mechanochemical) or combining it with acid leaching to achieve full silica–alumina valorization. Additionally, systematic techno-economic and life-cycle assessments are needed to inform practical deployment. Such strategic integration reflects broader research trends towards circular chemical use, reduced reagent footprints, and selective multi-component recovery.

While alkali leaching provides clear advantages over sintering and acid leaching, such as lower temperature operation, improved silica dissolution, and potential for reagent recycling, it is limited by the incomplete breakdown of crystalline minerals and the moderate alumina recovery without prior activation. Its main strength lies in efficiently converting silica into soluble silicates. To overcome these limitations and improve the extraction of both alumina and silica, alkali fusion was developed as a thermochemical extension of conventional alkali leaching.

### Alkali fusion

Alkali fusion is widely recognized as a highly effective technique for enhancing the chemical reactivity of crystalline minerals such as quartz (Lei et al. 2024). This method operates by breaking the stable Si–O–Si bonds and tetrahedral frameworks found in crystalline structures, thereby transforming inert phases into more reactive forms. During the fusion process, both amorphous and crystalline silica in CFA react with alkali sources to form water-soluble silicates. Specifically, refractory phases like quartz and mullite can be converted into more soluble compounds such as nepheline and noselite (Tang et al. 2019). This transformation significantly improves silica solubility in the subsequent leaching step.

Alkali fusion provides markedly higher silica extraction efficiencies than direct acid or alkaline leaching, particularly in CFA with low amorphous silica content (Su et al. 2011; Liu et al. 2016). This technique is therefore regarded as one of the most effective approaches for activating crystalline aluminosilicates. The process involves calcining the waste material with an alkali reagent (e.g., NaOH or Na₂CO₃) at elevated temperatures, typically between 500 and 900 °C. Under these harsh thermal conditions, the crystalline matrix is disrupted, enhancing the material’s reactivity (Lei et al. 2024). Supporting this, Li et al. (2024) demonstrated that using alkali fusion with additives such as Na₂CO₃, NaOH, NaCl, CaO, and CaCO₃ significantly enhanced the recovery of SiO₂ and Al₂O₃, yielding 2 to 5 times higher extraction efficiencies than conventional leaching methods. Similarly, Tang et al. (2019) found that rare earth element (yttrium, lanthanum, cerium, praseodymium, neodymium) recovery improved from 23% (via direct acid leaching) to 58% using alkaline fusion–leaching. Besides improving extraction efficiency, alkali fusion also accelerated leaching kinetics, reducing the time required to achieve equilibrium. In another study, Zhang et al. (2021) reported that alkaline fusion achieved a maximum silica conversion of 29%. Like other methods (alkali and acid leaching), the effectiveness of alkali fusion is governed by several key parameters, including the type and dosage of alkali, fusion temperature, reaction time, and the mass ratio of alkali to CFA. Thus, optimizing the interplay between temperature, alkali dosage, and time is essential for enhancing the reactivity, crystallinity, and leachability of key components like silicon and aluminum. Numerous studies have demonstrated that variations in these parameters lead to significant differences in mineral phase activation and elemental recovery. For instance, Kouamo et al. (2013) effectively activated natural volcanic ash using 70% NaOH at 550 °C. Li et al. (2024) explored a range of alkali agents, including NaOH and Na₂CO₃, across temperatures from 500 to 900 °C for CFA treatment. Tang et al. (2019) reported successful activation with Na₂CO₃ at 860 °C, while Xu et al. (2025) employed 50–67% NaOH at 850 °C to enhance CFA reactivity. A comparative assessment across studies indicates that fusion temperature and alkali dosage have synergistic effects. High temperatures and alkali content promote phase breakdown, but excessive conditions can form insoluble secondary phases that limit selective extraction, particularly for aluminum. These observations underscore the need for systematic optimization protocols. Table 7 summarizes key alkali fusion and acid-assisted fusion studies, highlighting the reagents, operational conditions, and achieved efficiencies.

**Table 7:** Overview of alkali fusion methods for alumina and silica recovery from CFA

Extraction system	Reagent concentration (M)	Optimal conditions	Efficiency (%)	References
Na₂CO₃–HCl (acid-assisted fusion)	3 M HCl	Fusion at 900 °C; 120 min acid leaching	Al₂O₃—98	Ji et al. (2007)
NaOH (solid-state fusion)	—	Fusion at 650 °C for 60 min; leaching for 240 min	SiO₂—29; Al₂O₃: 65	Zhang et al. (2021)
Alkali fusion–citric acid leaching	0.4 M citric acid	Fusion at 875 °C for 90 min; leaching at 90 °C for 1 h; S:L = 1:100	SiO₂—87; Al₂O₃—4	Li et al. (2024)
Alkali fusion–acid leaching	6 M HCl	Na₂CO₃ flux (CFA:Na₂CO₃ = 1:0.7); calcination at 880 °C for 1.5 h; leaching at 100 °C for 120 min; S:L = 1:6	Al—97	Zhao et al. (2025)

#### Effect of temperature

Some studies have underscored the temperature-dependent behavior of alkali fusion. For instance, Li et al. (2024) conducted alkali fusion experiments at temperatures ranging from 500 to 900 °C over 90 min and observed a consistent increase in total element leaching efficiency, from 42% at 500 °C to a peak of 70.09% at 875 °C. However, further increasing the temperature to 900 °C led to a decline in efficiency to 63%, indicating that excessively high temperatures can adversely affect extraction performance. In this study, element-specific trends were also observed as silicon recovery peaked at 875 °C. At the same time, aluminum extraction declined, likely due to the formation of stable sodium aluminosilicate phases that hinder Al dissolution. Elsewhere, Tang et al. (2019) explored fusion at 680 °C, 740 °C, 800 °C, and 860 °C for 30 min. At 680 °C, quartz remained largely unreacted, while at 860 °C, the formation of sodium silicate and nepheline was observed, accompanied by enhanced crystallinity and increased sodium aluminosilicate content. These findings emphasize that while elevated temperatures promote the breakdown of stable mineral phases and the formation of reactive silicates, excessive heat may lead to the formation of undesirable stable phases that limit extraction efficiency.

#### Mass ratio of alkali to CFA

Optimizing the alkali to CFA ratio enhances the dissolution of inert phases, promotes the formation of desirable mineral products, and improves the overall recovery of targeted elements. Several studies have shown that both insufficient and excessive alkali inputs can hinder reaction efficiency and product crystallinity. For example, Tang et al. (2019) reported that increasing the Na₂CO₃ to CFA ratio to 1:1.05 improved the disruption of CFA’s structure, enhancing reactivity. Similarly, Koshlak (2023) demonstrated that raising the NaOH to CFA ratio from 1:1.4 to 1:1.8 significantly improved the extraction of Si and Al. In another study, Xu et al. (2025) found that at a NaOH to CFA ratio of 1:1, the formation of synthetic aluminosilicate materials (zeolite A and X) was limited, as indicated by weak diffraction peaks. A ratio of 1.25:1 showed partial activation, with unreacted quartz and mullite remaining. At 1.5:1, strong crystallinity of zeolite A and X was achieved, marking it as the optimal ratio. However, increasing the ratio to 2:1 led to crystal aggregation and a decline in zeolite X intensity. Zhou et al. (2022) also examined the role of NaOH dosage in NaHS zeolite formation. At low ratios (0.5:1 and 1:1), activation was incomplete, resulting in minimal product formation. A 1.5:1 ratio initiated partial Si and Al extraction with some impurities, while a 2.0:1 ratio enabled complete activation and the formation of high-purity NaHS zeolite.

Although expressed differently, both the alkali to CFA mass ratio and the alkali concentration affect the availability of reactive alkali in the system. The mass ratio reflects the quantity of alkali relative to fly ash, while concentration determines the chemical strength of the solution. A high mass ratio with dilute alkali may be less effective than a lower ratio using a more concentrated solution. Lei et al. (2024) explored this relationship by varying NaOH concentrations (15%, 25%, 50%, and 75%) at 600 °C. At 15%, sodium aluminosilicate was the dominant Al-rich phase, due to preferential disruption of weaker Al–O bonds (512 kJ/mol) over stronger Si–O bonds (798 kJ/mol). At 25%, quartz began transforming into sodium aluminosilicate, sodium silicate, and sodium calcium silicate. At 50%, quartz content significantly decreased, with sodium aluminosilicate and sodium silicate dominating. By 75%, quartz was nearly eliminated, and sodium silicate prevailed, indicating advanced mineral breakdown. These findings highlight that both the alkali mass ratio and concentration work synergistically to enhance CFA activation, promote phase transformation, and improve the crystallization of target products. The studies collectively highlight that careful optimization of the alkali-to-CFA ratio and alkali concentration is critical for effective activation of CFA. Both insufficient and excessive alkali input can limit phase transformation, reduce crystallinity, or generate undesirable products. Optimal ratios and concentrations enable complete breakdown of inert phases, enhance the formation of targeted aluminosilicate or silicate products, and maximize Si and Al recovery. These findings underscore the importance of balancing chemical availability and reaction conditions, illustrating that synergistic control of both mass ratio and solution strength is key to achieving high-efficiency, selective CFA valorization.

#### Effect of fusion duration

Extended fusion time allows for prolonged interaction between alkali agents and CFA particles, facilitating deeper alkali penetration into the mineral matrix. This prolonged exposure enhances the breakdown of stable crystalline phases. Tang et al. (2019) demonstrated that increasing the fusion duration promotes the release of reactive silicon and aluminum species, as longer treatment allows more complete structural disruption. Short fusion periods (e.g., 1–2 h) typically affect only surface-bound or amorphous fractions of CFA, whereas longer durations (6–8 h) result in significant internal breakdown and higher yields of extractable elements. Similarly, Koshlak (2023) reported that extending the fusion time up to 6 h, under constant alkali dosage and temperature, lead to increased concentrations of soluble Si and Al in the leachate. After this point, no significant improvement was observed, as the reaction rate slowed down, a common feature of solid-state reactions nearing equilibrium. Lei et al. (2024) also noted that extended fusion times, particularly at higher NaOH concentrations (50–75%), facilitate more complete mineral transformation. This was evidenced by the disappearance of quartz and the formation of sodium silicate and aluminosilicate phases. These transformations require extended durations due to the high bond energies of Si–O (798 kJ/mol) and Al–O (512 kJ/mol) bonds. However, overly long fusion durations can introduce complications, such as particle sintering, increased energy consumption, and the formation of secondary or less soluble phases that hinder element recovery.

Further insight into this dynamic was provided by Li et al. (2024), who systematically investigated the impact of fusion time on mineral transformation and element leaching at elevated NaOH concentrations. Their findings revealed that at short fusion times (e.g., 15 min), moderate leaching occurred, extracting 63% of SiO₂ and 15% of Al₂O₃. When the fusion duration was extended to 90 min, silica extraction improved significantly to 86%, indicating enhanced activation and dissolution of silicon-bearing minerals. Interestingly, alumina extraction dropped sharply to 0.8% at 90 min, suggesting that extended fusion encouraged the formation of more crystalline, insoluble aluminosilicates that trapped alumina, reducing its availability in solution. This complex behavior underscores the importance of carefully optimizing fusion time. While longer durations favor silica dissolution, they may simultaneously hinder alumina recovery due to secondary phase formation, ultimately affecting the efficiency and selectivity of the alkali fusion process.

Alkali fusion bridges the gap between traditional sintering, direct acid leaching, and alkaline extraction. It overcomes the incomplete breakdown of refractory phases and moderate alumina recovery seen in alkaline leaching while offering higher overall oxide recovery than conventional methods. Yet, it still relies on high temperatures and produces solid residues. These limitations highlight the potential of combination methods, which can integrate multiple approaches to enhance efficiency, reduce energy use, and maximize selective recovery, setting the stage for hybrid extraction strategies.

Overall, alkali fusion, which combines thermal activation with chemical treatment, demonstrated substantial improvements in alumina recovery compared with conventional alkali leaching. By disrupting refractory phases such as mullite through high-temperature fluxing, alkali fusion produced more soluble intermediates, enhancing alumina extraction. Silica recovery was also enhanced in specific systems (up to 87%, Li et al. 2024), although the relative Al/Si ratio varied depending on the fusion conditions. Compared with simple alkali leaching, which improved silica dissolution but only partially extracted alumina, alkali fusion effectively overcame the structural limitations of CFA, providing a step-change in Al recovery. These findings highlight the potential of alkali fusion as a key intermediary method, bridging earlier alkali leaching approaches and more advanced combination strategies aimed at maximizing both alumina and silica yields while managing operational parameters.

### Combination methods

**Fig. 2:**
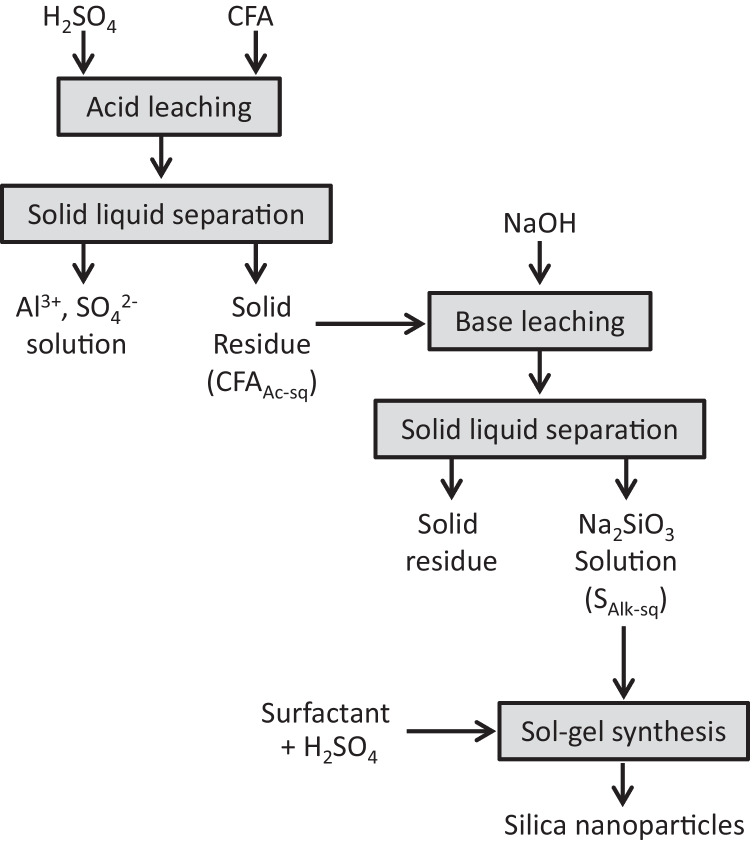
Schematic diagram of the sequential acid-alkaline leaching (Aphane et al. 2020)

While the two methods (direct alkaline and alkaline fusion) efficiently extract both silicon and aluminum from CFA, they often lead to aluminum contamination in the final sodium silicate solution, affecting its purity and industrial applicability. However, sequential acid-alkaline leaching (SAAL) offers a more selective approach by first extracting aluminum in an acid medium, leaving silicon intact for a subsequent alkaline extraction (Fig. 2). This method improves purity, reduces energy consumption, and ensures better control over extraction processes, making it a more efficient and cost-effective alternative to direct alkaline extraction and alkaline fusion. Compared to single-step methods, SAAL represents an integrated, selective approach that balances yield, purity, and operational feasibility.

This SAAL technique combines the benefits of both acid leaching and alkali dissolution, enhancing the efficiency of extracting valuable components from CFA. Initially, an acid leaching step (using HCl, H₂SO₄, or other acids) is used to remove unwanted impurities such as calcium, magnesium, and iron oxides. The resulting purified CFA residue is then subjected to alkali treatment, typically with sodium hydroxide (NaOH), to dissolve silica (Aphane et al. 2020). This approach maximizes the solubility of both silica (SiO₂) and alumina (Al₂O₃), providing a more balanced recovery of these key components, which are critical for various applications (Zhao et al. 2023).

The combined method has been found to offer significant advantages over single-step treatments, as it improves extraction yields, reduces processing time, and enhances the overall purity of the end products. Additionally, they contribute to more sustainable management of CFA by extracting multiple valuable components, making it a promising approach for the industrial reuse of this waste material. In a study by Guo et al. (2017), a combination method involving pre-desilication, Na₂CO₃ activation, and acid leaching was developed to enhance the simultaneous extraction of alumina and silica in CFA. The pre-desilication step, conducted using 20% NaOH at 100 °C for 2 h, dissolved approximately 37% of the SiO₂ content, effectively increasing the Al/Si molar ratio from 0.8 to 1.2. This adjustment proved critical in improving alumina extraction efficiency while significantly lowering the consumption of sodium carbonate. The same study continued to mix the desilicated CFA with raw CFA to achieve an Al/Si molar ratio of 1 mol Al per 1 mol Si; the process attained an Al₂O₃ dissolution rate of around 87%. It is imperative to note that this method also resulted in a 53% reduction in Na₂CO₃ usage compared to direct activation processes. This highlights the importance of controlling the Al/Si ratio and reagent consumption in combination methods to improve both economic and environmental performance. Thus, the study demonstrated that this integrated approach not only improves extraction efficiencies but also makes the process more economically and environmentally sustainable.

Other combination methods have gained attention for their ability to boost the recovery of both silicon and aluminum from CFA. For example, a study by Tang et al. (2019) investigated the extraction of rare earth elements from CFA through alkali fusion–acid leaching. The concentration of rare earth elements in the leachate after alkali fusion was more than twice that of direct leaching, with significantly improved leaching efficiency and a much faster leaching rate. Although their study focused on rare earth elements rather than aluminum and silicon, these findings are still relevant, as they highlight the effectiveness of combined methods in enhancing the extraction of elements from CFA. Li et al. (2024) found that the leeching rate of critical elements through direct organic acid (including tartaric acid, citric acid, and lactic acid) leeching was very low, ranging from 7 to 13%. However, adding alkali fusion with Na_2_CO_3_ to the extraction process improved the result significantly, with Na_2_CO_3_ fusion-citric acid leaching process being the highest, reaching 69%. Kumar et al. (2020) reported that extracting aluminum from CFA is challenging due to the high stability of the mullite phase, which resists both direct leaching and mechanical grinding. Their study showed that thermal treatment using NaOH, especially at 800 °C, effectively disrupted the mullite structure, allowing up to 83% of aluminum to be leached out when followed by hydrochloric acid treatment. They also demonstrated that microwave-assisted treatment provided a quicker and more energy-saving alternative, reaching 76% aluminum recovery in just 10 min. Overall, their combined thermal and acid leaching method proved efficient, cost-effective, and capable of yielding high-purity alumina (98% recovery), while silicon remained mostly intact, supporting selective extraction of aluminum. Wang et al. (2024) developed an effective method for separating and recovering silica and alumina from CFA through a sequence of reduction roasting, oxidation roasting, alkaline leaching, and Bayer digestion. During reduction roasting with Fe₂O₃, the aluminosilicate in CFA was broken down into hercynite (FeAl₂O₄) and cristobalite (SiO₂). The hercynite was then oxidized to hematite (Fe₂O₃) and alumina (Al₂O₃). Silica was efficiently extracted using sodium hydroxide leaching, while alumina was recovered through Bayer processing. This process achieved extraction rates of approximately 87% for silica and 90% for alumina, under optimal conditions. Overall, the study confirmed the success of this integrated route for high-yield recovery of alumina and silica from CFA. Elsewhere, Zhang et al. (2021) found that microwave-assisted hydrothermal extraction at 100 °C for 1 h achieved higher silica and alumina conversion than conventional heating for 3 h, due to rapid and uniform microwave heating. Silica conversion exceeded 19%, linked to amorphous SiO₂. In alkaline fusion, increasing the temperature from 550 to 650 °C boosted silica and alumina recovery by 22% and 65%, respectively, with further increases at 750 °C by 7% and 46%, respectively, confirming the temperature’s key role in enhancing extraction efficiency. Pan et al. (2023) conducted a mechanistic investigation into an integrated process combining NaOH treatment and citric acid leaching for the recovery of rare earth elements from coal fly ash. Their study showed that the sequential hydrothermal NaOH treatment dissolved approximately 60% of the ash, with about 75% of the total silicon and 60% of the total aluminum successfully leached from the original material. In another study, Ji et al. (2007) used alkali fusion, where CFA was calcined with soda at 900 °C prior to acid leaching. This process resulted in over 90% aluminum recovery. Across these studies, combination methods consistently outperform single-step methods in recovery efficiency, selectivity, and time efficiency, although energy consumption and reagent optimization remain critical challenges.

#### Operational factors influencing combined extraction techniques

Like the other extraction methods, the efficiency of combined methods is significantly influenced by several operational parameters, such as the solid-to-liquid (S/L) ratio. For instance, Li et al. (2024) investigated the effect of varying S/L ratios from 1:25 to 1:200 under fixed fusion and leaching conditions (875 °C alkali fusion, 0.4 mol/L citric acid, 50 °C, and 1-h leaching). Recovery efficiency peaked at an S/L ratio of 1:125, with 73% of critical elements extracted. At lower ratios, such as 1:25, recovery dropped to 54%, and further dilution beyond 1:125 led to a decline in leaching efficiency, likely due to reduced reagent contact per solid mass. Major element oxides were also tested, and the results showed relatively consistent SiO₂ content across all ratios (85 ± 2% to 88 ± 2%), whereas Al₂O₃ content varied, with the lowest residue value (0.7 ± 0.02%) at an S/L ratio of 1:150. Similarly, Tang et al. (2019) demonstrated the sensitivity of the process to alkali dosage and S/L ratios in rare earth element (REE) extraction from CFA. Increasing the Na₂CO₃-to-CFA mass ratio to 1:1.05 enhanced alkali activation due to intensified reactions, promoting complete structural breakdown. However, excessive alkali beyond this optimum (1:1.2 and 1:1.5) led to diminished REE leaching efficiencies (56% and 49%, respectively), likely due to secondary phase formation or saturation effects. The same study also highlighted that decreasing the S/L ratio from 1:5 to 1:20 improved REE recovery from 43 to 92%. Although more diluted systems led to lower REE concentrations in the leach liquor, the total amount extracted increased, underscoring the critical balance between reagent availability and solid loading. Pan et al. (2023) investigated the mechanism of an integrated process involving NaOH pretreatment followed by citric acid leaching for the recovery of rare earth elements from CFA. To assess the impact of NaOH concentration on silicon extraction, they employed NaOH solutions of 1 M, 2 M, 4 M, and 6 M. The highest silicon leaching rate was observed with 6 M NaOH, reaching approximately 47%, slightly higher than the 45% and 41% achieved with 4 M and 2 M solutions, respectively. Notably, the maximum silicon removal using 2 M and 4 M NaOH was comparable, but both began to decline gradually after 100 min of leaching. These findings collectively emphasize that careful optimization of the solid-to-liquid ratio and reagent concentration is crucial for maximizing the dissolution of valuable components in combined extraction processes. Both reagent overuse and insufficient dosing, as well as inappropriate slurry concentrations, can hinder reaction completeness and resource efficiency, thereby limiting the overall feasibility and scalability of these methods.

#### Synthesis

Combination methods marked a significant advancement in CFA valorization, building on sintering, direct acid leaching, and alkali treatment. Unlike lime sintering, which achieved high alumina recovery but required large CaO inputs and high temperatures, and unlike single-step acid or alkali leaching, which were limited in silica or alumina extraction, these approaches integrated sequential or simultaneous treatments to enhance both SiO₂ and Al₂O₃ recovery. Methods such as NaOH pre-desilication followed by Na₂CO₃ activation and acid leaching, sequential acid–alkaline leaching (SAAL), and thermal/microwave-assisted alkali–acid treatments improved disruption of refractory phases, optimized Al/Si ratios, and enhanced mass transfer, yielding alumina recoveries of 76–87% and silica recoveries of 37–89% as shown by Table 8. Nevertheless, challenges remain in energy efficiency, reagent optimization, and achieving maximal simultaneous recovery. These limitations highlight the need for alternative approaches that can achieve high selectivity and recovery under milder conditions, paving the way for hydrothermal treatments, which offer controlled phase transformation, lower temperatures, and potential for scalable, environmentally sustainable extraction.

**Table 8:** Combination methods for alumina and silica recovery from CFA

Extraction system	Reagent concentration (M or wt% or g)	Optimal conditions	Efficiency (%)	References
Pre-desilication + Na₂CO₃ activation + acid leaching	20 wt% NaOH; Na₂CO₃ + acid	Pre-desilication at 100 °C for 120 min; Na/Al molar ratio = 1; S:L = 1:1	SiO₂—37; Al₂O₃: 87	Guo et al. (2017)
Sequential acid–alkaline leaching (SAAL)	5 M H₂SO₄; 10 M NaOH	Acid leaching at 95 °C for 240 min; alkaline leaching at 95 °C for 60 min	SiO₂—89; Al₂O₃: 82	Aphane et al. (2020)
Thermal + acid leaching (NaOH–HCl)	NaOH (solid) + 1 M HCl	NaOH activation at 800 °C for 60 min; acid leaching at 80 °C for 60 min	Al—83	Kumar et al. (2020)
Microwave-assisted alkali–acid leaching	NaOH + HCl	Microwave at 100 °C for 10 min	Al—76	Kumar et al. (2020)
Reduction–oxidation roasting + low-temperature alkaline leaching and Bayer digestion	120 g NaOH	10:3:1 CFA: Fe₂O₃: charcoal mix; reduction and oxidation roasting at 1100 °C for 120 min; alkaline leaching at 90 °C for 120 min; Bayer digestion at 260 °C for 60 min	SiO₂—87; Al₂O: 90	Wang et al. (2024)

### Hydrothermal treatments

While combination methods have shown improved efficiencies in extracting alumina and silica from CFA, recent attention has also turned toward hydrothermal treatments as a viable alternative or complementary route. Unlike high-temperature sintering or fusion methods, hydrothermal techniques typically operate under moderate thermal and pressure conditions, offering a more energy-efficient and environmentally benign approach. Hydrothermal treatment, particularly the one-step hydrothermal process, has proven to be an effective method for recovering alumina and silica from CFA, as it facilitates the dissolution of both amorphous and crystalline aluminosilicate phases (Shoppert et al. 2017). This process not only enhances the leaching efficiency of alumina and silica into solution but also promotes the formation of valuable secondary phases such as zeolites, thereby adding further value to the treated CFA (Shoppert et al. 2017). Furthermore, the process consumes less sodium hydroxide (NaOH), generates lower volumes of waste, and yields a purer alumina solution suitable for downstream applications like pseudo-boehmite synthesis (Cao et al. 2021). In a typical hydrothermal process, CFA is mixed with an alkaline agent (most commonly NaOH) and subjected to temperatures (150–250 °C) and pressures above atmospheric levels. These conditions accelerate the breakdown of the CFA matrix, particularly facilitating the dissolution of silica into soluble sodium silicate. The treatment is especially effective for CFA with low amorphous silica content, as the hydrothermal conditions help deconstruct crystalline mineral phases and enhance silica release (Su et al. 2011). After the reaction, the mixture is filtered to recover the sodium silicate solution, while the remaining solids may be discarded or further utilized.

Cao et al. (2021) developed a one-step hydrothermal extraction process combining sodium hydroxide (NaOH) and lime (CaO) to enhance alumina recovery from coal fly ash. Conducted at 210 °C for 3 h with 30 g/L NaOH and a CaO/SiO₂ molar ratio of 1:1. The process achieved a 60% Al_2_O_3_ leaching efficiency. In this system, silicon was immobilized as porous tobermorite mineral, which facilitated separation by preventing silica from re-dissolving into the leachate. However, part of the aluminum became incorporated into undesirable aluminiferous zeolite phases, slightly diminishing overall recovery. Despite this limitation, the method offers significant advantages over conventional Bayer and sintering–leaching routes by operating under milder conditions and accommodating lower Al/Si ratios in feedstocks. Similarly, Xing et al. (2024) demonstrated the viability of a mild alkali hydrothermal process for the simultaneous leaching of Al₂O₃ and SiO₂. Their method achieved extraction rates of approximately 42% for alumina and 41% for silica. Optimal alumina recovery occurred under moderate conditions (≤ 180 °C and ≤ 2 h), beyond which the formation of phases like hibschite diminished efficiency (Xing et al. 2024). Furthermore, alumina recovery peaked at 40% at 180 °C (1 h) and 36% at 170 °C (2 h), while silica extraction remained below 1%. Notably, an increased Ca/Si ratio promoted alumina leaching but also favored hibschite formation (Xing et al. 2024). In another study, Chao et al. (2023) developed a one-step hydrothermal method to convert CFA into silicon–potassium fertilizer while extracting alumina. At 200 °C with 280 g/L K₂O for 1 h, they achieved 27% Al₂O₃ extraction and 35% SiO₂ content. Another successful extraction was reported by Li et al. , who explored the extraction of alumina from CFA by the mixed-alkaline (NaOH + Ca(OH)_2_) hydrothermal method. Through this process, the alumina extraction ratio reached 91%. It is imperative to note that the hydrothermal behavior of alumina and silica in CFA differs considerably. Alumina, typically present as mullite, dissolves more readily under hydrothermal conditions, forming soluble aluminate ions. Silica, mainly found as quartz or silicate minerals, is less soluble and tends to react with calcium to form stable solids like tobermorite. This selective dissolution pathway not only enhances alumina extraction but also transforms silicon into a valuable by-product, facilitating a more holistic and environmentally favorable utilization of CFA in industrial processes. Table 9 summarizes recent studies on the hydrothermal treatment of CFA.

**Table 9:** Summary of hydrothermal treatment conditions and recovery efficiencies for CFA

Extraction system	Reagent concentration (M or wt%)	Optimal conditions	Efficiency (%)	References
Mixed-alkali hydrothermal	40 wt% NaOH	260 °C; 45 min; S:L = 1:12	Al₂O₃—91	Li et al. ()
One-step hydrothermal process	0.75 M NaOH	210 °C; 180 min	Al₂O₃—60	Cao et al. (2021)
NaOH hydrothermal conversion	5.0 M NaOH	100–200 °C; 180 min	SiO₂—33–67; Al₂O₃—13–23	Zhang et al. (2021)
K₂O-based one-step process	3.0 M K₂O	200 °C; 60 min S:L = 1:4	SiO₂—35; Al₂O₃—27	Chao et al. (2023)
Mild alkali hydrothermal	7.5 M NaOH	100–180 °C; 120 min; S:L = 1:5; Ca/Si = 0.8	SiO₂—34; Al₂O₃—40	Xing et al. (2024)

Like other extraction methods, hydrothermal treatment for the recovery of alumina and silica from CFA is influenced by critical operational parameters such as temperature, alkaline concentration, and the liquid-to-solid (L/S) ratio. For instance, Chao et al. (2023) observed that increasing the hydrothermal leaching temperature from 120 to 240 °C, at a constant K₂O concentration of 280 g/L, enhanced the Al₂O₃ extraction rate from 8 to 29%. This improvement was attributed to the increased reactivity of KOH at elevated temperatures (Table 9). However, overall alumina recovery remained constrained due to the formation of stable potassium aluminum silicate compounds, which limit Al leaching. Similarly, Zhang et al. (2021) reported partial conversion of amorphous SiO₂ and Al₂O₃ during hydrothermal treatment between 100 and 200 °C, achieving silica and alumina recoveries of 33–67% and 13–23%, respectively. Supporting this, Querol et al. (2002) found that temperatures ranging from 125 to 200 °C promoted enhanced leaching of both aluminum and silicon. Further improvements were reported by Li et al. (), who showed that increasing the reaction temperature from 230 to 280 °C led to a significant rise in alumina extraction, reaching 91% at 260 °C, with high extraction maintained even at higher temperatures. Alkaline concentration also plays a pivotal role; for example, Chao et al. (2023) demonstrated that increasing the K₂O concentration from 240 to 360 g/L boosted Al₂O₃ recovery, particularly under elevated temperatures. For example, at 160 °C, recovery increased from 9 to 23%, and at 200 °C, from 25 to 29%, due to enhanced molecular activation and reaction kinetics. In addition, the liquid-to-solid (L/S) ratio significantly affects alumina recovery. Under a fixed NaOH concentration of 40 wt%, Li et al. found that increasing the L/S ratio led to a sharp rise in Al extraction, peaking at 91% when the ratio was below 12, beyond which gains became more gradual. From the results, it is apparent that hydrothermal treatments offer a recovery of both alumina and silica from CFA, addressing the selectivity limitations of single-step acid, alkali, or fusion-based methods. They operate under relatively mild conditions, with moderate energy and chemical requirements, reduced waste generation, and the potential for forming valuable secondary phases such as zeolites. However, recovery efficiencies remain moderate compared to high-yield processes like alkali fusion or combined methods, indicating that standalone hydrothermal approaches are insufficient for maximizing the efficiencies. These characteristics suggest that hydrothermal methods are well-suited as part of a combination or sequential strategy, where they can complement acid or alkali pre- or post-treatments to improve overall extraction, enhance selectivity, and reduce environmental impact. Therefore, future research should focus on optimizing operational parameters, integrating hydrothermal processes with other extraction techniques, and evaluating their techno-economic and environmental performance to fully exploit their potential in sustainable CFA valorization.

### Synthesis and perspective

CFA valorization has evolved from traditional sintering, which prioritized alumina recovery but is energy- and lime-intensive. As the next evolutionary process, direct acid leaching maintained the focus on Al while eliminating lime and operating under milder conditions, though large acid volumes limited scale-up. Enhanced acid leaching and assisted methods (pressure, microwave, sinter–acid, lime) accelerated kinetics and improved Al yields but required costly equipment and corrosion control. The field then moved toward alkaline leaching and alkali fusion, enabling co-recovery of both Si and Al or primarily Si, respectively; fusion excels at breaking refractory phases but increases energy and chemical demands. Combination and sequential methods integrate acid and alkali steps to maximize recovery and selectivity of both elements, while hydrothermal treatments provide environmentally benign, energy-efficient solutions even for low-reactivity CFA. Overall, research shifted from single-step, Al-focused, high-energy processes toward multi-step, selective, and sustainable strategies that balance high recovery, energy efficiency, and environmental performance (Table 10). While earlier methods, such as lime sintering or acid leaching, achieved high alumina recoveries, they often neglected silica recovery and involved high energy or reagent consumption. Modern combination approaches have improved the simultaneous extraction of both alumina and silica, offering more balanced recoveries, yet most studies report only moderate overall efficiencies and remain limited in scale or scope. These limitations highlight the need for alternative methods, such as hydrothermal treatments with combined methods, which can enable selective, high-yield extraction under milder and potentially more sustainable conditions.

**Table 10:** Comparison of various extraction methods for Al₂O₃/Al and SiO₂/Si recovery

Evaluation criteria	Sintering processes	Direct acid leaching	Enhanced acid leaching	Alkaline leaching	Alkali fusion	Combination/sequential methods	Hydrothermal treatments
Recovery (%)	Al₂O₃/Al: 60–98	Al₂O₃/Al: 25–94	Al₂O₃/Al: 70–90	SiO₂/Si: 28–71; Al₂O₃/Al: 32–72	SiO₂/Si: 29–87; Al₂O₃/Al: 4–98	SiO₂/Si: 37–89; Al: 76–90	SiO₂/Si: 33–67; Al₂O₃/Al: 40–91
Temperature (°C)	900–1200	95–230	80–1200	65–150	500–900 °C	50–900	120–280
Reaction time (minutes)	60–120	60–360	30–240	60–240	15–480	10–120	60–180
Process efficiency	Efficient in breaking refractory phases (e.g., mullite); enhances Al leachability	Simple setup; selectively dissolves Al, leaves Si intact; effective for amorphous Al	Pressure-, microwave-, sinter–acid-, and lime-assisted methods enhance kinetics, surface reactivity, and reduce leaching time	Operationally simple and low-energy; enables co-recovery of Si and Al without high thermal input; best for amorphous CFA	Excellent silica recovery from crystalline CFA; high overall dissolution efficiency	Integrates acid and alkali steps for higher yield and selectivity; balances purity and reagent use; reduces reagent use	Dissolves amorphous + crystalline aluminosilicates; moderate energy input; suited for low-reactivity CFA
Feasibility/scalability	High energy and reagent demand (e.g., lime) and costly	Large acid volumes hinder scale-up; silica co-dissolution forms gelatinous residues that impede filtration and reduce recovery	Elevated temperature and acid concentration raise cost and corrosion risk	Secondary phases and high slurry viscosity limit aluminum recovery and scalability	Energy-intensive (500–900 °C), and reactions can form secondary phases that reduce selectivity for aluminum and silica	Multi-step process with reagent handling increases complexity and cost, limiting large-scale use	High-pressure equipment, long reaction times, and operational costs may limit scale-up
Environmental/technological implications	Generates Ca-silicate residues; high lime use raises carbon and waste footprint	Medium-higher acid concentrations boost extraction but increase handling, effluent, and neutralization needs	Requires acid recovery; microwave and pressure lower chemical load, while sinter–acid and lime raise waste and carbon footprint	High alkali use requires effluent neutralization and the risk of scaling	Uses large amounts of Na₂CO₃/NaOH, leading to high carbon footprint and chemical waste	Lowers chemical use; shorter processing reduces energy, but some steps remain energy- and time-intensive with solid residues	Environmentally benign; minimal secondary waste; energy-efficient under optimized conditions
Overall insight	Research has shifted from lime-only to hybrid sintering, improving selectivity and yield while reducing lime use, though energy costs remain high	Compared to sintering, direct acid leaching operates under milder thermal conditions with lower energy demand, achieving high Al recovery and better selectivity over silica, eliminates lime use, but generates higher effluent loads	Compared to sintering and direct acid leaching, enhanced leaching speeds up extraction and improves yields, but requires costly equipment and higher operational costs	Enables integrated Si–Al recovery; is more sustainable than acid leaching, but yields are limited by crystalline CFA	Alkali fusion is less eco-friendly but more effective at breaking refractory phases, achieving high silica recovery and extraction efficiency, though with lower aluminum selectivity	Outperforms single-step methods in yield and selectivity, promising for circular resource recovery	Energy-efficient and clean; attractive for sustainable CFA valorization when combined with other methods
Key references	Verbaan & Louw (1989); Bai et al. (2011); Yao et al. (2014); Aphane et al. (2015); Doucet et al. (2016)	Verbaan and Louw (1989); Gudyana et al. (1992); Bai et al. (2011); Li et al. (2011); Wu et al. (2012); Yao et al. (2014); Aphane et al. (2015); Sangita and Panda (2016)	Matjie et al. (2005); Bai et al. (2011); Wu et al. (2012); Ma et al. (2021)	Aphane et al. (2020); Faraji et al. (2022); Collins et al. (2023); Xing et al. (2024); Widowati et al. (2024); Rosita et al. (2024)	Tchakoule Kouamo et al. (2013); Tang et al. (2019); Li et al. (2024); Xu et al. (2025)	Ji et al. (2007);Tang et al. (2019); Kumar et al. (2020); Pan et al. (2023); Li et al. (2024); Wang et al. (2024)	Querol et al. (2002); Li et al. (2014a, b); Zhang et al. (2021); Chao et al. (2023); Li et al. (2024); Xing et al. (2024)

## CFA utilization for high-value products

Following the successful extraction of silica and alumina from CFA, considerable attention has been directed toward the conversion of these recovered components into high-value nanomaterials such as silica (SiNPs) and alumina nanoparticles (AlNPs) including the synthesis of zeolites. These CFA-derived products offer unique physicochemical properties, including high surface area, thermal stability, and chemical reactivity, which make them valuable for a wide range of industrial and environmental applications (Aphane et al. 2020; Oktavia et al. 2023; Saleh 2024).

### Silica nanoparticles (SiNPs) derived from sodium silicate

**Fig. 3:**
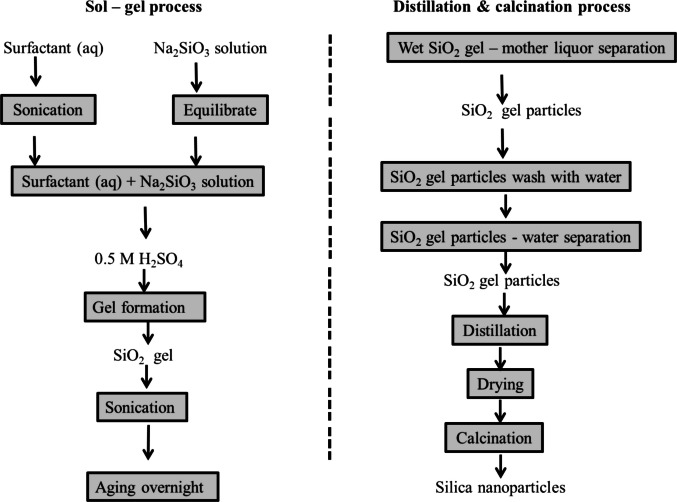
Process flow diagram for the preparation of silica nanoparticles from CFA-derived sodium silicate solutions

Once sodium silicate is extracted from coal fly ash via a leaching process, typically by dissolving the ash in sodium hydroxide and resulting with Na_2_SiO_3_ solution after filtration, the synthesis of silica nanoparticles proceeds through several key steps. The Na_2_SiO_3_ solution is first neutralized with an acid, such as hydrochloric or sulfuric acid, to trigger hydrolysis and condensation reactions that form a silica gel (Imoisili and Jen 2024; Aphane et al. 2020). The gel is then aged under controlled conditions (e.g., overnight at 55 °C) to enhance its structural integrity (Yadav and Fulekar 2019; Aphane et al. 2020). After aging, it is separated from the liquid phase by centrifugation or filtration and washed thoroughly with deionized or warm water or even ethanol to remove residual ions like Na⁺ and SO₄^2^⁻ (minerals and impurities) (Yadav and Fulekar 2019; Imoisili and Jen 2024). In some protocols, a solvent exchange using n-butanol is applied to reduce drying stress. The final drying is followed by calcination at high temperatures (e.g., 650 °C) to eliminate remaining organics, yielding pure, amorphous silica nanoparticles. Some variations exist, such as the drying temperatures, aging time, and the use of surfactants like polyethylene glycol (PEG) to control particle morphology (Aphane et al. 2020). However, the core methodology consistently involves sodium silicate extraction, gelation, purification, and thermal treatment. The processes of preparing silica nanoparticles from CFA via the sol–gel method are displayed in Fig. 3.

Researchers such as Aphane et al. (2020) demonstrated the successful synthesis of high-purity (up to 99 wt% SiO₂) mesoporous silica nanoparticles from South African CFA using Na₂SiO₃ solutions obtained via SAAL and DAL processes. Similarly, McCondochie et al. (2023) reported the production of high-purity silica nanoparticles from CFA-derived sodium silicate obtained via NaOH leaching. In their study, a 35 wt% yield of SiO₂ nanoparticles was achieved from an initial SiO₂ content of 51 wt%, indicating efficient recovery and transformation into nanoscale material. In another study, amorphous nanosilica was synthesized and characterized from South African CFA, achieving a yield of 30% for the amorphous silica nanoparticles (ASNPs) with 99% purity after extraction (Imoisili and Jen 2024). Yadav and Fulekar (2019) investigated the green synthesis and characterization of amorphous silica nanoparticles derived from CFA, reporting a purity of up to 94% in the final product. Similar studies also report the synthesis of these silica nanoparticles (Imoisili and Jen 2022; Yadav et al. 2023; Singh et al. 2024). While numerous studies globally have successfully demonstrated the synthesis of high-purity silica nanoparticles from CFA, there remains a significant research gap within Africa, particularly in South Africa. Despite the abundant availability of coal fly ash as a raw material, local research on its valorization into silica nanomaterials is limited compared to other regions such as Asia and Europe. Addressing this geographical disparity is crucial to unlocking the potential of South Africa’s CFA resources for sustainable nanomaterial production. Expanding research efforts in this area will not only contribute to environmental waste management but also foster innovation in applications like catalysis, water treatment, and nanofertilizers, ultimately supporting regional development and circular economy goals (Yadav et al. 2023; Saleh 2024; Singh et al. 2024).

### Alumina nanoparticles (AlNPs)

Alumina (Al₂O₃) nanoparticles can be synthesized from aluminum sulfate solutions obtained from CFA through thermochemical treatment using ammonium sulfate ((NH₄)₂SO₄). For example, McCondochie et al. (2023) achieved a 31 wt% yield of alumina nanoparticles relative to the initial 34 wt% Al₂O₃ content in South African CFA, indicating a relatively efficient conversion. However, the study also noted that the purity of the nanoparticles required improvement, particularly through optimized washing steps to reduce residual sulfur content. Efforts to synthesize alumina from aluminum sulfate solutions generated by leaching CFA with sulfuric acid (H₂SO₄) have been hindered by extremely low pH values (below zero), which complicate the synthesis process.

Although several studies have investigated the extraction and recovery of aluminum from CFA, most of them focus on using the recovered aluminum or alumina for applications such as ceramics, refractories, and other bulk materials. However, these studies do not typically advance toward the synthesis of alumina nanoparticles. This creates a notable gap in the literature, as the potential to convert aluminum extracted from CFA into high-value alumina nanoparticles remains largely underexplored. To date, most alumina nanoparticles reported in the literature are synthesized from high-purity, aluminum-rich commercial precursors rather than waste-derived sources like CFA. Considering the wide range of applications for alumina nanoparticles, including use in catalysts, microelectronics, biomedical coatings, adsorbents, and thermally resistant materials, further research is needed to explore CFA as a viable, low-cost, and sustainable precursor for CFA-derived alumina nanoparticles (Yadav and Fulekar 2020). Such an approach could not only reduce production costs but also promote waste valorization and support circular economy initiatives by transforming industrial byproducts into value-added nanomaterials.

### Aluminosilicate for zeolite synthesis

**Fig. 4:**
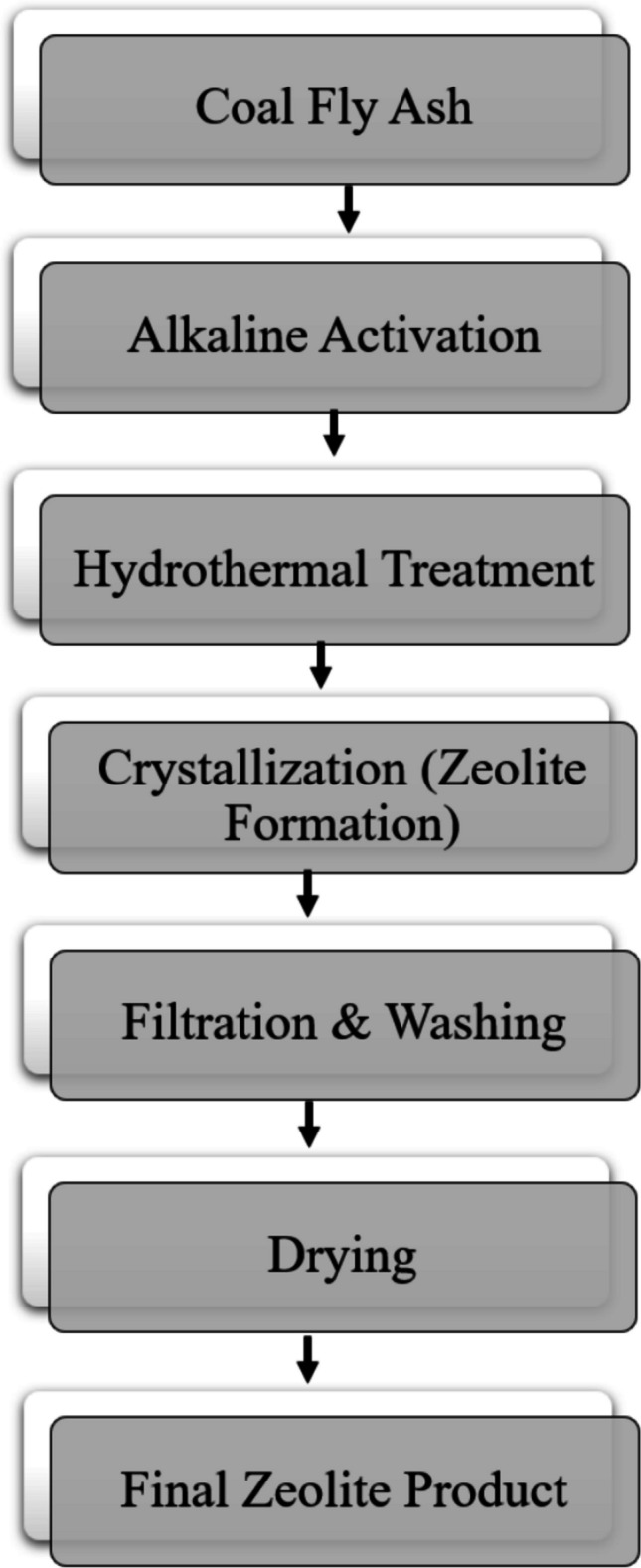
Schematic representation of zeolite synthesis from fly ash

Aluminosilicates extracted from coal fly ash have been widely studied for their potential to synthesize various types of zeolites with broad industrial and environmental applications (Musyoka et al. 2012; Mainganye et al. 2013; Brassell et al. 2016; Makgabutlane et al. 2020; Miricioiu and Niculescu 2020; Chen et al. 2023a, b; Manyepedza et al. 2024; Fitriani et al. 2025). Typically, the synthesis involves alkaline activation of fly ash using NaOH or KOH, followed by hydrothermal treatment at elevated temperatures (90–160 °C) for several hours to days (Hong et al. 2017; Xu et al. 2025; Koshlak 2023). The resulting gel undergoes crystallization, yielding zeolite phases such as Na-P1, faujasite, or sodalite, depending on synthesis conditions like Si/Al ratio, temperature, pH, and aging time (Ferdov 2020; Ma et al. 2023). Post-synthesis steps include filtration, washing, and drying to isolate the final zeolite product (Brassell et al. 2016; Hong et al. 2017; Koshlak 2023; Ma et al. 2023). The overall synthesis process of zeolites from fly ash is illustrated in Fig. 4. Zeolites, including zeolite X, zeolite Y, zeolite A, ZSM-5, zeolite P (including Na-P1), K-chabazite, and K-phillipsite, have proven effective in multiple fields. They are commonly used as catalysts in petroleum refining processes like hydrocarbon cracking and isomerization, as well as in chemical synthesis (Kumar et al. 2013; Primo and Garcia 2014). In environmental applications, they serve as efficient adsorbents and ion exchangers in water treatment for the removal of heavy metals and ammonium ions (Koshlak 2023). Their high surface area and porous structures also make them suitable for gas adsorption and separation, including carbon dioxide capture and methane storage (Boer et al. 2023). Furthermore, these zeolites are utilized in soil remediation and other catalytic processes, underscoring the value of CFA as a low-cost and sustainable source for producing functional aluminosilicate materials.

## Challenges, research gaps, and future perspectives

Analyses across studies reveal that hybrid or mixed extraction methods consistently outperform traditional sintering and single-step acid or alkaline leaching, achieving both alumina and silica recoveries under milder conditions. Moreover, energy-efficient techniques, such as pressurized or microwave-assisted hydrothermal processes, have shortened reaction times from several hours to as little as 10–60 min while requiring less chemical input, demonstrating clear advantages for scalability and industrial feasibility. However, several gaps remain. Critical key parameters such as leaching temperature, extraction time, solid-to-liquid ratio, and reagent are not fully optimized. Moreover, most extraction studies remain highly system-specific, with process conditions tailored to particular CFA compositions and experimental setups. This lack of standardization limits the scalability and industrial application of lab-developed methods. As a result, industries continue to rely on expensive commercial precursors, such as MCM-type mesoporous materials, despite CFA being a promising, low-cost alternative. Another pressing issue is the geographical concentration of research efforts. The bulk of studies have been conducted in countries like China, India, the USA, and Russia, while regions such as Africa, particularly South Africa, a major coal-dependent country, are underrepresented. This geographic gap hinders the adaptation of technologies to local CFA compositions and resource constraints.

To close these gaps, future research should prioritize the following.

1. Systematic optimization of extraction parameters across a broader range of CFA sources
2. The development of environmentally friendly and cost-effective leaching systems, particularly those based on organic acids, which is a promising area of research. Biodegradable acids such as citric and tartaric acid offer significant advantages over conventional inorganic acids due to their low environmental impact and sustainability. For instance, Li et al. (2024) demonstrated that citric acid not only minimizes ecological risks during and after leaching but also achieves high extraction efficiency, with up to 89% of SiO₂ recovered at a concentration of 0.2 mol/L.
3. Region-specific and comparative studies that evaluate low-cost, locally available reagents
4. Greater emphasis on separation selectivity, purity, and waste minimization
5. Above all, interdisciplinary and applied research is urgently needed to support the practical implementation of CFA valorization technologies and contribute to global sustainable development goals.
6. Future perspectives should also include the integration of extraction with downstream product synthesis, such as high-purity silica, alumina nanoparticles, or zeolite production, with quantitative targets for yield and purity. For example, target benchmarks of > 90% SiO₂ and Al₂O₃ recovery and > 95% product purity could provide measurable objectives for process development. Additionally, research should prioritize life cycle assessment (LCA) and techno-economic analysis (TEA) to ensure environmental sustainability and commercial viability, which will bring scientific novelty and practical guidance to the field.

## Conclusion

Comparative evidence indicates that traditional sintering and single-step acid or alkaline leaching selectively extract alumina while leaving silica largely unreacted, limiting overall utilization of CFA. The introduction of alkali and alkali fusion techniques improves these early methods by enhancing silica dissolution and partially mobilizing alumina; however, they often rely on high energy input, excessive reagent consumption, and generate environmentally burdensome residues requiring neutralization. In response, combined and sequential extraction approaches, particularly alkali fusion–acid leaching and similar hybrids, have achieved more balanced recovery of both SiO₂ and Al₂O₃, with efficiencies commonly reaching 80–90%. Nevertheless, these methods still face scalability and environmental challenges. So, recent attention has shifted toward hydrothermal treatments, which operate under comparatively milder conditions and lower chemical demand, offering a more sustainable route for simultaneous Al–Si recovery. Yet, current recoveries remain moderate, which suggests that hydrothermal methods alone may not yet rival the extraction performance of multi-step systems but hold significant promise as part of integrated, low-impact process designs. Therefore, the future of CFA valorization lies in the strategic coupling of hydrothermal processes with hybrid/combined extraction schemes, guided by systematic optimization of parameters such as reagent concentration, liquid-to-solid ratio, reaction temperature, and residence time. Furthermore, establishing standardized performance metrics for recovery efficiency, energy consumption, and environmental footprint will be essential for scaling these methods from laboratory to industrial application. By integrating efficiency with environmental stewardship, CFA can transition from an abundant waste product into a sustainable secondary resource, supporting circular economy goals and low-carbon material production.

## Data Availability

This article is a review of previously published studies. All supporting data are available in the cited publications.
